# Methylation-GC-MS/FID-Based Glycosidic Linkage Analysis of Unfractionated Polysaccharides in Red Seaweeds

**DOI:** 10.3390/md22050192

**Published:** 2024-04-24

**Authors:** Barinder Bajwa, Xiaohui Xing, Stephanie A. Terry, Robert J. Gruninger, D. Wade Abbott

**Affiliations:** Lethbridge Research and Development Centre, Agriculture and Agri-Food Canada, 5403 1st Avenue South, Lethbridge, AB T1J 4B1, Canada; barinder.bajwa@agr.gc.ca (B.B.); xiaohui.xing@agr.gc.ca (X.X.); stephanie.terry@agr.gc.ca (S.A.T.); robert.gruninger@agr.gc.ca (R.J.G.)

**Keywords:** methylation analysis, reductive hydrolysis, whole food fiber, whole cell wall, anhydro sugar, agarose, carrageenan, sulfated galactan, mixed linkage xylan, principal component analysis

## Abstract

Glycosidic linkage analysis was conducted on the unfractionated polysaccharides in alcohol-insoluble residues (AIRs) prepared from six red seaweeds (*Gracilariopsis* sp., *Prionitis* sp., *Mastocarpus papillatus*, *Callophyllis* sp., *Mazzaella splendens*, and *Palmaria palmata*) using GC-MS/FID analysis of partially methylated alditol acetates (PMAAs). The cell walls of *P. palmata* primarily contained mixed-linkage xylans and small amounts of sulfated galactans and cellulose. In contrast, the unfractionated polysaccharides of the other five species were rich in galactans displaying diverse 3,6-anhydro-galactose and galactose linkages with varied sulfation patterns. Different levels of cellulose were also observed. This glycosidic linkage method offers advantages for cellulose analysis over traditional monosaccharide analysis that is known for underrepresenting glucose in crystalline cellulose. Relative linkage compositions calculated from GC-MS and GC-FID measurements showed that anhydro sugar linkages generated more responses in the latter detection method. This improved linkage workflow presents a useful tool for studying polysaccharide structural variations across red seaweed species. Furthermore, for the first time, relative linkage compositions from GC-MS and GC-FID measurements, along with normalized FID and total ion current (TIC) chromatograms without peak assignments, were analyzed using principal component analysis (PCA) as a proof-of-concept demonstration of the technique’s potential to differentiate various red seaweed species.

## 1. Introduction

Red algae (Rhodophyta) is the oldest phylogenetic division of eukaryotic algae, comprising approximately 5000 species that are primarily marine macrophytes known as red seaweeds [1]. Some red seaweed species have been harvested and used as food by humans for over 2800 years [2]. Red seaweeds contain various polysaccharides, including, but not limited to, Floridean starch, cellulose, xylan, neutral mannan, sulfated mannan, and sulfated galactan [1,3]. Galactans, the major polysaccharide components of the majority of red seaweeds, consist of linear chains composed of alternating 3-linked and 4-linked residues and include three types: carrageenan-type, agar-type (e.g., agarose, porphyrans), and d/l hybrids of the two types, differing in the following structural features: presence or absence of sulfate substitution, presence or absence of anhydro sugars, and absolute configurations (d, l) of monosaccharides [4]. Sulfated galactans, such as carrageenans, are bioactive carbohydrates and functional additives with many reported health-enhancing properties [5,6,7,8]. These polysaccharides are mass-produced as stabilizers, emulsifiers, homogenizers, and encapsulating materials widely used in the food, pharmaceutical, and other industries [4,9,10]. Two main types of xylans, both homoxylans, have been identified in red algae: the 1,3-β-d-xylans, found as microfibrils in the cell walls of *Porphyra umbilicalis* (Bangiales), and the mixed-linkage 1,3;1,4-β-d-xylans, present in the matrix phase of cell walls in the orders Nemaliales and Palmariales [3]. Given the diversity of natural structures and variations in the abundance of polysaccharides in red seaweeds, analyzing the chemical composition of these polysaccharides is crucial for understanding their functions and for their optimal application in seaweed-based industries.

GC-MS/FID-based linkage analysis is a fundamental tool that provides convincing evidence of the types and compositions of monosaccharide linkages present in polysaccharides, oligosaccharides, and biomacromolecules that contain carbohydrate moieties [11,12,13,14,15,16,17]. This process involves permethylating the free hydroxyl groups in polysaccharides, followed by hydrolysis to release partially methylated monosaccharides, reduction to C-1 deuterium-labeled alditols via sodium borodeuteride (NaBD_4_) treatment, then peracetylation to yield partially methylated alditol acetate (PMAA) derivatives for GC-MS/FID analysis [13,18,19]. Conducting linkage analysis on unfractionated cell walls is crucial for understanding the relative compositions of all polysaccharides within the samples, as this approach eliminates the need for fractionation and the potential loss of specific polysaccharides [20]. The procedure has been frequently used for analysis of unfractionated polysaccharides in the form of alcohol-insoluble residues (AIRs) prepared from higher plants and fungi, whose cell walls mainly consist of neutral monosaccharide, uronic acid, and amino sugar linkages [21,22,23,24,25,26,27]. However, the procedure cannot be directly applied to the linkage analysis of unfractionated polysaccharides of red seaweeds due to the presence of sulfated and/or anhydro sugar linkages in galactans. This necessitates special treatments to prevent the under-methylation of sulfated galactans due to their poor solubility in dimethyl sulfoxide (DMSO) in their inorganic salt forms, the loss of methylated sulfated galactans during product cleanup, or the degradation of 3,6-anhydro-galactose during acid hydrolysis [19], as detailed in Section 2.1.

This study aimed to improve the conventional methylation-GC-MS/FID method to determine the glycosidic linkage compositions of unfractionated polysaccharides in six red seaweed species of commercial interest.

## 2. Results and Discussion

### 2.1. Adaptation of the Method for Unfractioned Polysaccharides of Red Seaweed

#### 2.1.1. Permethylation

Sulfated polysaccharides in their inorganic salt forms are poorly dissolved in DMSO and need to be cation-exchanged to their triethylammonium (TEA) salt form for gaining solubility in DMSO. This process is achievable through either dialysis against a deionized water solution of triethylammonium chloride (TEAC) or passing through a cation exchange column (TEA salt form). The dialysis method was found superior for sulfated galactans [15,19]; however, it is not feasible for unfractionated polysaccharides of red seaweeds because, during dialysis, galactans dissolve in water and separate from water-insoluble polysaccharides (e.g., cellulose). This results in the final freeze-dried samples having polysaccharides with varying water solubilities non-homogeneously distributed, making it impossible to aliquot the dialyzed samples for subsequent methylation. Consequently, all samples recovered from the same dialysis tubing must be permethylated together, with the small sample amount constrained by the permethylation process’s capacity. Dialyzing small amounts of samples in separate dialysis tubing increases sample loss and is more inconvenient, labor intensive, costly, and time consuming than dialyzing relatively large amounts and then aliquoting for linkage analysis, a common practice for whole cell wall analysis of higher plants [13]. Furthermore, the inability to aliquot effectively and the requirement to use small amounts of starting material in each reaction tube make it difficult to analyze anhydro sugar linkages, which requires aliquoting one portion of the same permethylated sample for TFA hydrolysis and retaining other identical samples from the same tube for reductive hydrolysis. Additionally, there is no evidence to disprove that sulfated polysaccharides may be trapped or encapsulated by insoluble cell wall residues, hindering their dissolution in water and full conversion to TEA salt form during dialysis prior to methylation.

Unfractionated polysaccharides, containing crystalline polysaccharides such as cellulose, need to be well suspended in DMSO for a successful methylation reaction. This is usually achieved by magnetically stirring the sample in heated DMSO (e.g., 60 °C) [13,28]. It was observed in this study that finely ball-milled dry AIR powders formed a virtually homogeneous suspension in DMSO at 60 °C with magnetic stirring overnight, in contrast to the observation that the powders of sulfated polysaccharide standards (listed in Section 3.1.3) remained intact without disassembly or disintegration when subjected to the same treatment in DMSO. We do not believe this observed difference has not been previously reported. The following mechanisms are proposed for future studies to examine. In dry AIR powder, sulfated galactans are associated with other polysaccharides to form cell walls and extracellular matrix structures [29]. Heated DMSO dissolves non-sulfated polysaccharides from the powder, while sulfated galactans remain in the powder due to their poor solubility in DMSO in their inorganic salt forms. The loss of the non-sulfated polysaccharides weakens the structure and integrity of the powder, causing its breaking into many tiny particles by the physical shearing force of magnetic stirring in heated DMSO. These particles are very small and not easily visible to the naked eye, which results in the appearance of a virtually homogeneous suspension. Additionally, this removal of non-sulfated polysaccharides by DMSO may result in the small pieces from disassembled powder being very porous (with the empty space previously occupied by the non-sulfated polysaccharides), and the porous particles have a relatively large surface area in DMSO, facilitating contact with reactants for methylation. In contrast, powders of purified sulfated polysaccharides, in their inorganic salt forms, form a complex resistant to DMSO extraction due to their poor solubility in DMSO. Consequently, the powder remains unaltered by magnetic stirring, staying visibly intact in DMSO.

**Scheme 1:**
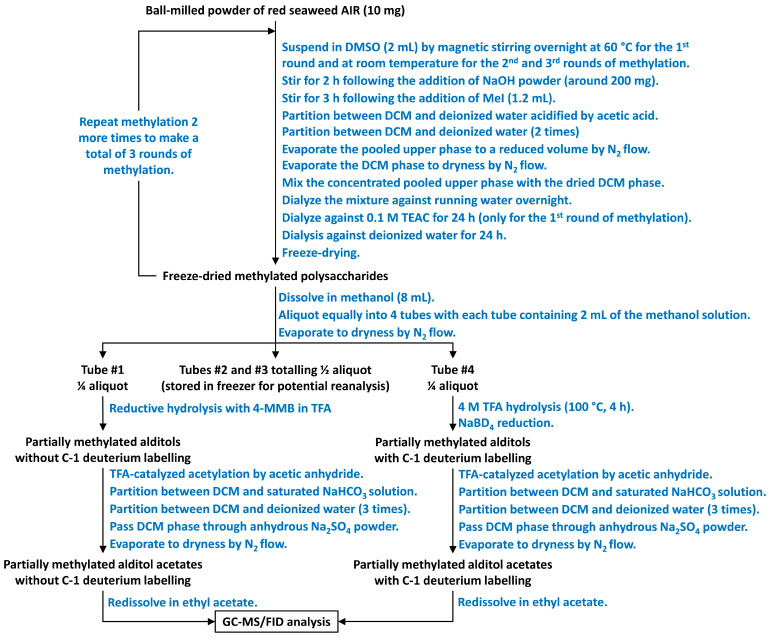
Flow diagram showing the steps of the preparation of PMAAs from unfractionated polysaccharides in ball-milled powder of red seaweed AIR.

Polysaccharides can be methylated by methyl iodide (MeI) in DMSO in the presence of strong bases such as sodium hydride [30,31,32,33], potassium hydride [34], n-butyllithium [35], potassium tert-butoxide [36], and sodium hydroxide (NaOH). The Ciucanu procedure, permethylation by MeI in DMSO with freshly prepared NaOH powder [18], has been improved and proven suitable for methylating polysaccharides [28], especially whole cell walls that contain crystalline polysaccharide structures [13]. This procedure was used in the current study. Satisfactory methylation was obtained for crystalline cellulose even with only one round of methylation (Figure S1). Following this first methylation, the sample, with sulfated galatans partially methylated, underwent TEAC dialysis during product cleanup, as depicted in Scheme 1 and detailed in Section 3.3.1. An overnight magnetic stirring in DMSO at room temperature then allowed the complete dissolution of the partially methylated galactans (in TEA salt form) and the other polysaccharides for the second round of methylation. During the cleanup step after the second methylation, the product was not subjected to TEAC dialysis. As expected, the methylated sulfated galactans, despite being in their inorganic salt forms, easily dissolved in DMSO with magnetic stirring at room temperature overnight. Apparently, the presence of methyl groups enhanced their solubility in DMSO. An additional round of methylation was conducted to ensure the complete methylation of free hydroxyl groups in the polysaccharides, with the sample dissolved in DMSO by magnetic stirring at room temperature and the TEAC dialysis step omitted as well (Scheme 1). This method was applied to permethylate ~10 mg of polysaccharides in a single glass reaction tube for analytical purposes. The fully methylated polysaccharides dissolved easily in methanol, making it very convenient to aliquot the resulting solution into multiple glass tubes for further experiments.

It is critical to remove MeI during the product cleanup after each methylation to avoid related side reactions during the subsequent acid hydrolysis, reduction, and peracetylation, and most importantly, for the protection of the analysts, as MeI is highly toxic [37]. Stevenson and Furneaux (1991) added water into the permethylated reaction product and then bubbled N_2_ through the mixture until the visual disappearance of cloudiness caused by the presence of MeI, followed by dialysis of the mixture [19]. The same N_2_ sparging method was also used to remove MeI from permethylated heparan disaccharide before cleanup with a C18 SPE cartridge [38]. In another report on cleaning up permethylated fucoidan, partitioning between chloroform and acidified deionized water was conducted, followed by the evaporation of the lower chloroform phase containing the MeI to dryness and combining the water phase with the dried lower phase for subsequent dialysis to remove sodium salts and residual DMSO [39]. In the current study, the latter procedure was improved by using dichloromethane (DCM) to replace chloroform for the partitioning, based on the previous report that replacing chloroform with DCM in the extraction of per-*O*-methylated carbohydrates significantly reduced degradation products to below 0.5%, a reduction that could be further minimized by neutralizing the reaction mixture with acid before partitioning [40]. Furthermore, as detailed in Section 3.3.1, the collected pooled water phases from the multiple partitionings were evaporated to reduce the volume by half under a flow of N_2_, provided by a generator, to ensure the removal of any minor levels of MeI that may exist in the upper water phase, before pooling the water phase with the dried lower phase for dialysis. It is important to note that both the water phase and the DCM phase from the partitioning need to be collected because permethylated neutral polysaccharides are soluble in DCM and thus stay in the lower phase, while permethylated sulfated galactans are water-soluble and, therefore, remain in the upper phase.

#### 2.1.2. Depolymerization and Reduction

**Figure 1:**
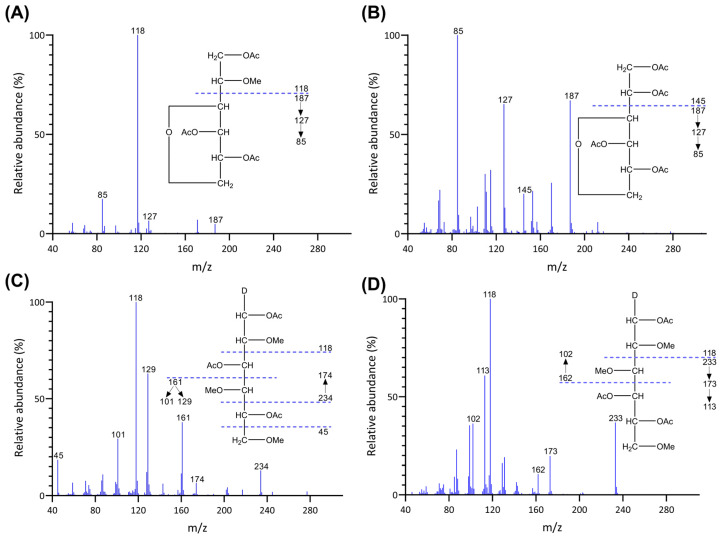
EI-MS spectra and ion fragmentation patterns of PMAAs from (**A**) 4-AnGal*p*, (**B**) 2,4-AnGal*p*, (**C**) 3-Gal*p*, and (**D**) 4-Gal*p* of galactans of *Callophyllis* sp.

Permethylated polysaccharides from higher plant cell walls and fungi are typically depolymerized by trifluoroacetic acid (TFA) hydrolysis (e.g., 2 M TFA at 120 °C or 4 M TFA at 100 °C) into monosaccharides that are further reduced by NaBD_4_ to alditols [13,41]. However, the anhydro sugar 3,6-anhydro-galactose in red seaweed galactans can be destroyed by TFA hydrolysis [19]. Therefore, a reductive hydrolysis process was developed, employing acid-stable 4-methylmorpholine borane (4-MMB) to reduce the 3,6-anhydro-galactose monosaccharides released by acid hydrolysis to alditols, thus protecting them from degradation [19]. Because 4-MMB was used instead of NaBD_4_ for the reduction, the resulting alditols were not C-1 deuterium labeled. Consequently, alongside the reductive hydrolysis, it is necessary to perform standard TFA hydrolysis on another aliquot of the same sample, followed by NaBD_4_ reduction to generate C-1 deuterium-label alditols for non-anhydro sugars, as detailed in Section 3.3.2. This facilitates peak identification based on the electron impact mass spectrometry (EI-MS) ion fragmentation patterns of non-anhydro sugar linkages in unfractionated polysaccharides of red seaweeds. Examples are shown in Figure 1.

Conventional GC-based monosaccharide analysis tends to underestimate crystalline cellulose in unfractionated cell walls due to difficulties in the initial acid hydrolysis stage in quantitatively releasing monosaccharides from the crystalline structure. Crystalline cellulose is resistant to TFA hydrolysis, necessitating the use of stronger acids, such as sulfuric acid, for its effective decrystallization and depolymerization to glucose [20]. Despite the availability of microscale monosaccharide analysis techniques that involve neutralizing and removing sulfuric acid with dioctylamine in chloroform [13], and the Blackeney method for macroscale monosaccharide analysis [42], sulfuric acid hydrolysis remains less convenient and can result in less clean GC chromatograms compared to TFA hydrolysis. Furthermore, the more destructive nature of sulfuric acid compared to TFA can lead to the degradation of monosaccharides released from cell walls, adversely affecting the quantitative quality of the analysis [43]. However, while native crystalline cellulose is resistant to TFA hydrolysis, permethylation before TFA hydrolysis allows the decrystallization of cellulose, facilitating its depolymerization by TFA and enabling reliable GC-MS/FID analysis of released glucose [44,45]. In the current study, crystalline cellulose from the unfractionated polysaccharides of red seaweeds was decrystallized and methylated using an improved Ciucanu procedure [18,28], enabling the quantitative release of partially methylated glucose by 4 M TFA hydrolysis for conversion into PMAAs for GC-based quantitation.

#### 2.1.3. Peracetylation and Final Cleanup

Alkaline acetylation, involving the use of base catalysts like pyridine or sodium acetate, became popular for its ability to avoid artifact formation, a common issue with preliminary acid-catalyzed acetylation (e.g., the use of sulfuric acid) [46,47]. Borate, resulting from the reduction with NaBD_4_, forms stable borate esters with vicinal diol groups under alkaline conditions, inhibiting the acetylation process and necessitating the removal of remaining borates as volatile trimethyl borates through a tedious and time-consuming process of repeated evaporation to dryness with methanolic acetic acid and then with absolute methanol [13]. Voiges et al. (2012) reported that acidic acetylation conditions, utilizing a TFA and acetic anhydride mixture at a 1:5 ratio (*v*/*v*) at 60 °C, not only yielded results comparable to alkaline-catalyzed acetylation with high recovery rates and reproducibility but also significantly simplified the process by eliminating the need to remove borate through repeated co-evaporation in methanolic acetic acid and methanol [48]. Therefore, the TFA-catalyzed acetylation was used in this study. After evaporation of the reaction mixture to dryness under N_2_, the residual TFA was neutralized and removed by partitioning between saturated NaHCO_3_ and DCM. The residual NaHCO_3_ was further removed by additional three rounds of partitioning between deionized water and DCM, as detailed in Section 3.3.3. The DCM phase was then filtered through a glass wool-clogged Pasteur pipette packed with sodium sulfate (Na_2_SO_4_) to remove residual water, a method that has been proven effective by previous reports [41,49], before evaporation to dryness and reconstitution in ethyl acetate for GC-MS/FID analysis. Ethyl acetate was chosen over DCM as the solvent for the final PMAAs due to having lower toxicity and being a greener chemistry [50,51].

#### 2.1.4. Identification and Quantitation of PMAAs

A 60-m medium polarity SP-2380 column was used for the efficient separation of the PMAAs with enhanced separation capability compared to a 30-m column of the same type. The identification of PMAAs was performed by comparing their retention times and EI-MS ion fragmentation patterns against PMAA references prepared from polysaccharide standards and methyl glycosides. The quantitation of PMAAs was achieved through the combined use of GC-MS and GC-FID. Using EI-MS, pairs of C-1 deuterium-labeled PMAAs derived from 2-Xyl*p* and 4-Xyl*p*, as well as 2,3,6-Gal*p* and 2,4,6-Gal*p*, can be distinguished despite their structural symmetry making them otherwise indistinguishable chromatographically [13]. Moreover, calculating the molar ratio for each pair from their extracted ion chromatograms enables the splitting of their shared peak area, thereby allowing the acquisition of respective TIC or FID peak areas to quantify each PMAA in the pair separately [25]. Without C-1 deuterium labeling, the EI-MS spectra of PMAAs in each pair became indistinguishable, necessitating conventional TFA hydrolysis-NaBD_4_ reduction alongside reductive hydrolysis, where deuterium labeling is not applicable, on the whole red seaweed cell wall. It is worth mentioning that there has been a preference for GC-MS-based quantitation in the analysis of PMAAs from unfractionated higher plant whole cell walls [24,26,27]. This method often uses relative peak area or, more commonly, relative molar percentages, assuming a direct proportionality between each PMAA’s quantity and ratio of its TIC peak area to its molecular mass, according to a widely accepted protocol [13]. However, red seaweed whole polysaccharides, with fewer and less varied PMAA peaks compared to their higher plant counterparts, allow easier GC-FID peak assignments using comprehensive PMAA standards. FID offers superior quantitation capabilities compared to EI-MS, due to its higher linear dynamic range [52]. The GC-FID-based quantitation benefits from utilizing FID response factors calculated based on the effective carbon number concept and verified by Sweet et al. (1975) [53]. These FID response factors were applied to all PMAAs except 3,6-anhydro-galactose linkages in the current study. The FID response factor for 4-AnGal*p* was experimentally determined to be 0.49 from methylation analysis of an agarose standard, applying a 1:1 molar ratio of 4-AnGal*p* to 3-Gal*p*, with 3-Gal*p* having a response factor of 0.74 [53]. Based on the response factor of 4-AnGal*p*, the response factor for 2,4-AnGal*p* was calculated as 0.54, according to the report that substituting a methyl group at the *O*-2 position with an acetyl group increases the relative response factor by 0.05 [53]. These values were lower than the response factors of 0.64 for 4-AnGal*p* and 0.72 for 2,4-AnGal*p* reported by Stevenson and Furneaux (1991) [19]. The discrepancy might be attributed to their use of the Hakomori methylation method, which is milder than the Ciucanu method and better avoids degradation [40,54,55], such as the oxidation of uronic acids [56], even though the Ciucanu method has been proven essential for the permethylation of crystalline polysaccharides in whole cell walls [13,20,28]. However, the greater degradation of 3,6-anhydro-galactose linkages with the Ciucanu methylation compared to the Hakomori methylation is not a significant technical issue, as the degradation margin can be offset by applying lower response factors based on experimental determination.

**Figure 2:**
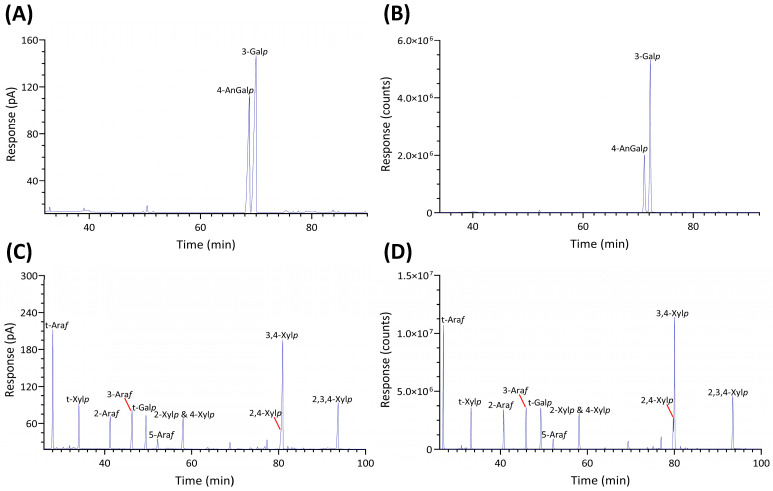
GC chromatograms of PMAAs prepared from agarose and corn fiber xylan standards, including (**A**) GC-FID and (**B**) GC-TIC chromatograms of agarose-derived PMAAs, alongside (**C**) GC-FID and (**D**) GC-TIC chromatograms of PMAAs from corn fiber xylan.

Significant differences were observed in the TIC and FID responses to PMAAs from 3,6-anhydro-galactose linkages. This discrepancy is highlighted by the notably smaller relative peak areas of 3,6-anhydro-galactose linkages in the GC-TIC chromatogram than in the GC-FID chromatogram of the identical sample solution from the same GC vial. Conversely, the relative peak areas of non-anhydro sugar PMAAs appeared similar across both detections. An example of such difference is demonstrated with PMAAs from agarose and corn fiber xylan standards in Figure 2A,B. The observed discrepancy in the response of AnGla*p* linkages between EI-MS and FID detection cannot be attributed to chromatographic factors, since identical columns were used for PMAA separation in both methods and the quantitatively normal detection of non-anhydro sugar PMAAs was achieved without any chromatographic issues (Figure 2C,D). This pointed to the difference being detector related. During the GC-MS experiments, the standard ionization energy of 70 eV for EI ionization was used, suggesting that this ionization energy might be suboptimal for ionizing 3,6-anhydro-galactose PMAAs and could lead to low ionization efficiency. It is recommended to investigate the effects of ionization energy on the recovery rate of 3,6-anhydro-galactose PMAAs and to optimize for the best ionization efficiency in future studies. Notably, a recent study calculated the EI-MS response factors for linkages of 4-AnGal*p* and 2,4-AnGal*p* relative to their neutral sugar counterparts, based on experimental linkage analysis of κ-carrageenan and ι-carrageenan standards, thereby enabling the reliable determination of seaweed samples containing these linkages by GC-MS [57].

### 2.2. Results of Linkage Compositions of Unfractionated Polysaccharides in Six Red Seaweeds

**Figure 3:**
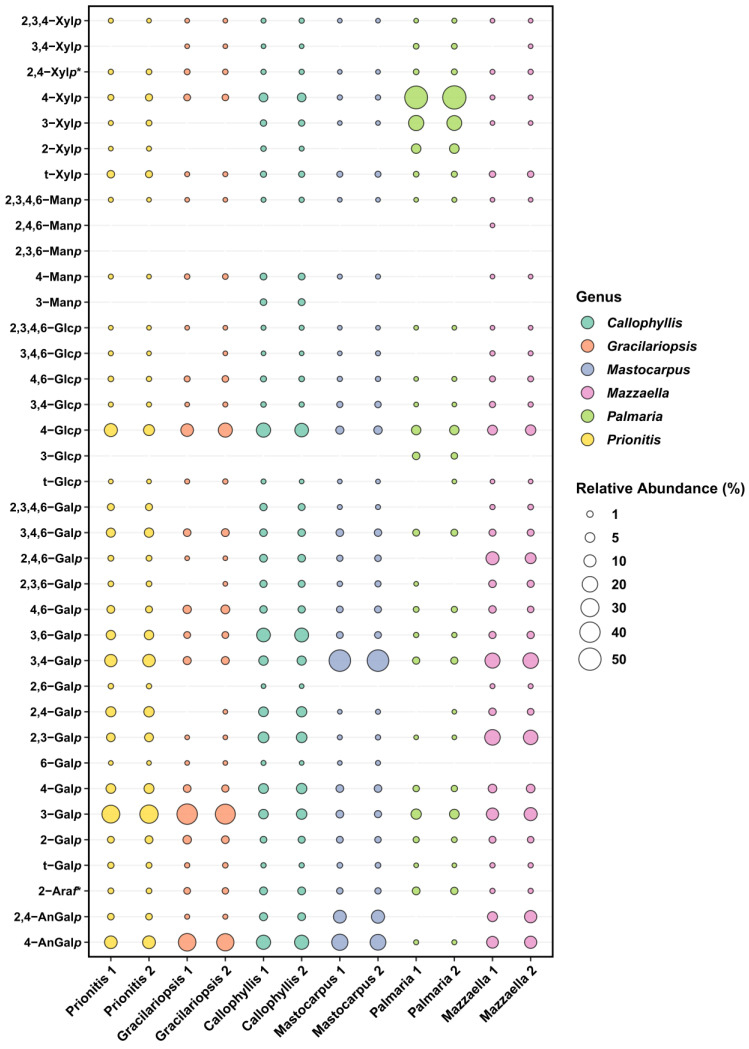
Bubble plot showing the relative linkage composition (Mol%) quantified by GC-FID from each of the two separate experiments conducted on unfractionated polysaccharides of six red seaweed species.

**Figure 4:**
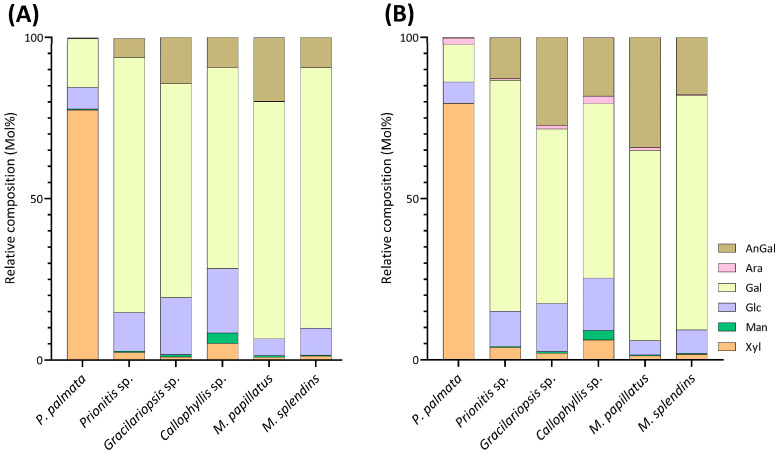
Relative monosaccharide compositions (Mol%) of unfractionated polysaccharides calculated by summing up all the linkage compositions, quantified by (**A**) GC-MS and (**B**) GC-FID, for each monosaccharide of six red seaweed species. AnGal, 3,6-anhydro-galactose; Ara, arabinose; Gal, galactose; Glc, glucose; Man, mannose; Xyl, xylose. Two separate experiments were conducted.

**Figure 5:**
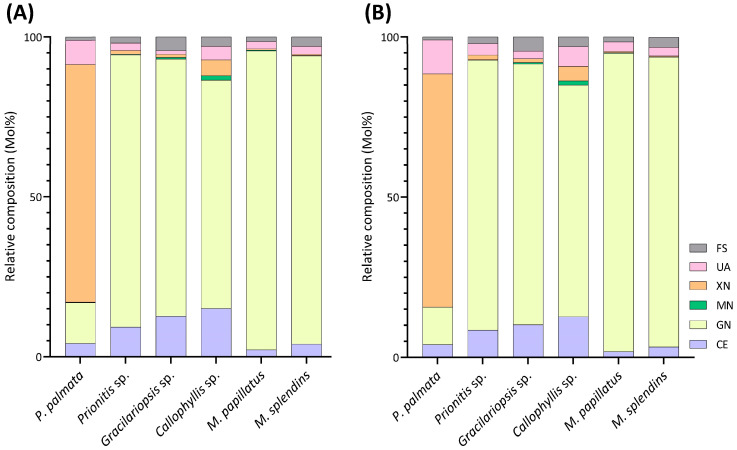
Estimated polysaccharide compositions (Mol%) calculated by summing up compositions of all the linkages, quantified by (**A**) GC-MS and (**B**) GC-FID, assigned to each type of polysaccharide of six red seaweed species. UA: unassigned linkages; XN: xylan; MN: mannan; GN: galactan; CE: cellulose; FS: Floridean starch. Two separate experiments were conducted.

**Figure 6:**
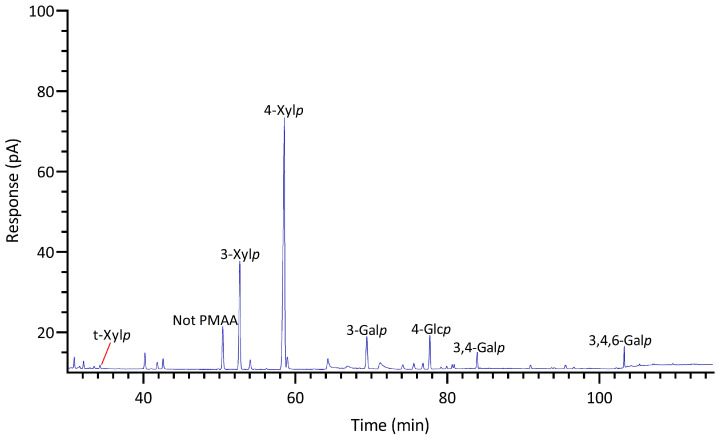
GC-TIC chromatogram of PMAAs from the glycosidic linkages in unfractionated polysaccharides of *Palmaria palmata*.

**Figure 7:**
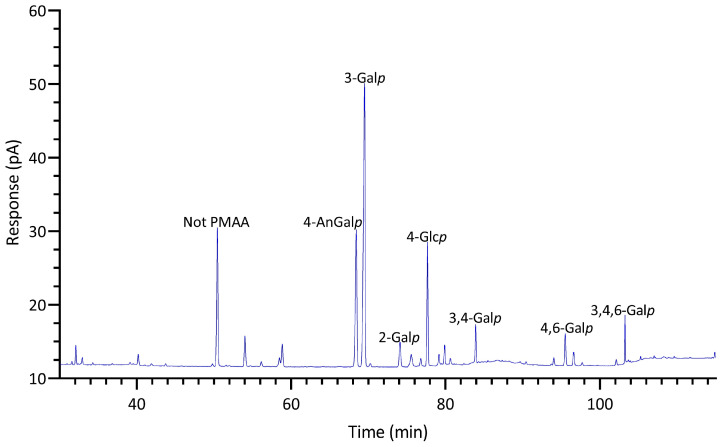
GC-TIC chromatogram of PMAAs from the glycosidic linkages in unfractionated polysaccharides of *Gracilariopsis* sp.

**Figure 8:**
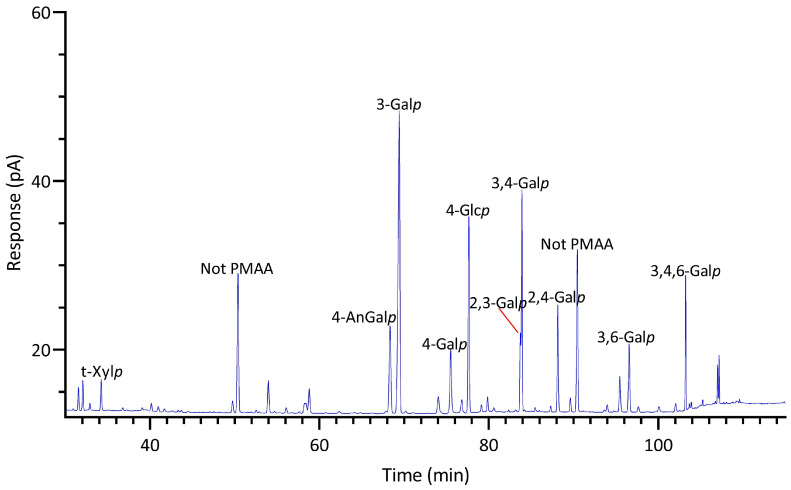
GC-TIC chromatogram of PMAAs from the glycosidic linkages in unfractionated polysaccharides of *Prionitis* sp.

**Figure 9:**
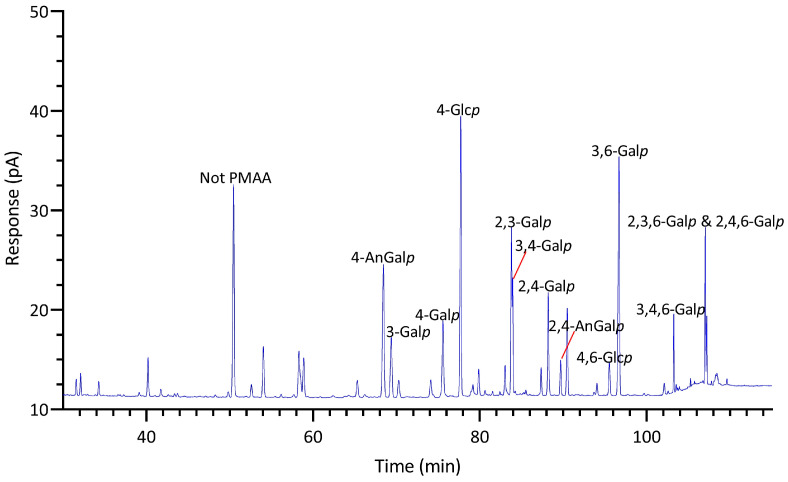
GC-TIC chromatogram of PMAAs from the glycosidic linkages in unfractionated polysaccharides of *Callophyllis* sp.

**Figure 10:**
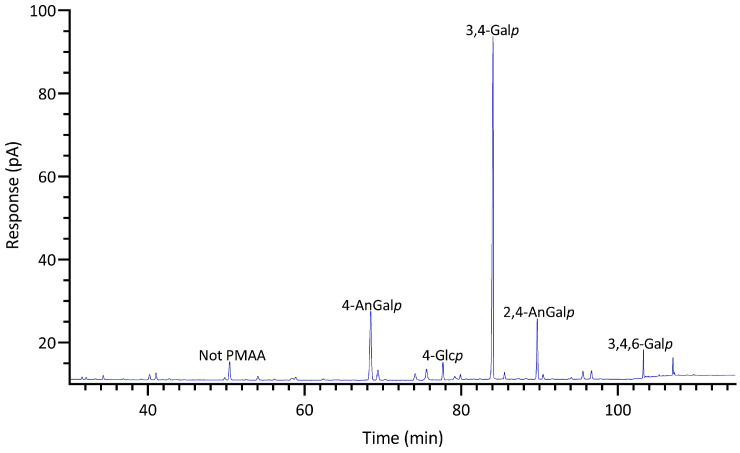
GC-TIC chromatogram of PMAAs from the glycosidic linkages in unfractionated polysaccharides of *Mastocarpus papillatus*.

**Figure 11:**
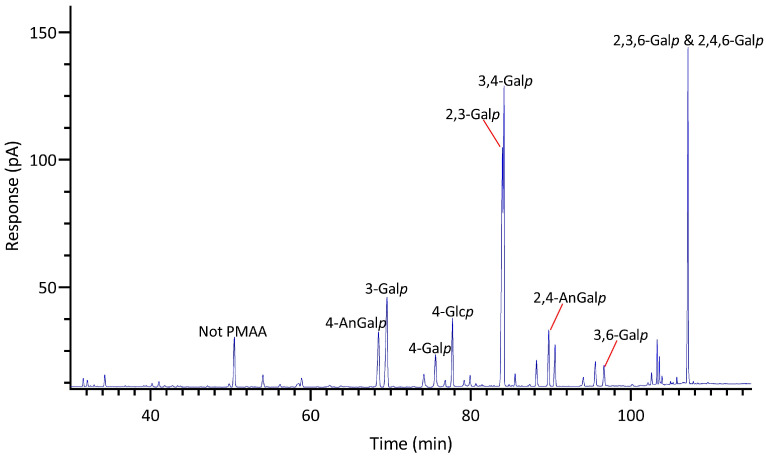
GC-TIC chromatogram of PMAAs from the glycosidic linkages in unfractionated polysaccharides of *Mazzaella splendins*.

The linkage analysis of unfractionated polysaccharides in six red seaweed species revealed a total of 38 unique linkages across all species studied. No rhamnose or fucose linkages were detected in any red seaweed, indicating no detectable contamination from green and brown seaweeds. Relative linkage compositions along with standard deviations calculated from two separate experiments conducted on each seaweed AIR are presented in Table 1. The relative compositions obtained from each of the two separate experiments are presented as a bubble blot (Figure 3), demonstrating similar results for linkage data from the two experiments. This indicates that the separate experiments conducted on each seaweed AIR yield reproducible results. The relative monosaccharide compositions, calculated by summing up all the linkage values from each monosaccharide, are presented in Figure 4 and Table S1. The polysaccharide compositions, estimated based on the relative linkage compositions, are presented in Figure 5 and Table S2. As discussed in Section 2.1.4, much lower relative compositions of 2-AnGal*p* and 2,4-AnGl*p* were observed using GC-MS than with GC-FID. Therefore, all linkage values presented below in Section 2.2.1, Section 2.2.2, Section 2.2.3, Section 2.2.4, Section 2.2.5 and Section 2.2.6 were from GC-FID quantitation. Linkage results calculated based on GC-MS are included in the Supplementary Document. GC-FID chromatograms of the PMAAs prepared from the six species are shown separately in Figure 6, Figure 7, Figure 8, Figure 9, Figure 10 and Figure 11.

#### 2.2.1. *Palmaria palmata*

Unlike most red seaweeds, *P. palmata*, belonging to the order Palmariales, is notable for its rich content of 1,3;1,4-β-d-xylan, making it a targeted species for 1,3;1,4-β-d-xylan extraction [58]. The AIR of *P. palmata* was analyzed to reveal compositions of 18% 3-Xyl*p* and 54% 4-Xyl*p* (Table 1, Figure 6). Xylose accounted for 80% of the overall monosaccharide composition (Figure 4, Table S1). The estimated compositions of the AIR included approximately 73% 1,3;1,4-β-d-xylan, followed by 12% galactans, with minor amounts of cellulose and Floridean starch (Figure 5, Table S2), which was in good agreement with a previous report [59], indicating that the cell walls of *P. palmata* were primarily composed of 1,3;1,4-β-d-xylan. Minor levels of 2,4-Xyl*p* and 3,4-Xyl*p* were detected. Notably, a low level of 3,4-Xyl*p* has been previously reported in 1,3;1,4-β-d-xylan extracted from *P. palmata* [60]. The identification of 2,4-linked and 3,4-linked xyloses suggested possible branching or substitutions, supported by previous reports that 1,3;1,4-β-d-xylan in *P. palmata* might form covalent bonds with sulfated and/or phosphorylated xylogalactoproteins [61,62]. The 4-Xyl*p* to 3-Xyl*p* ratio in 1,3;1,4-β-d-xylan from *P. palmata* varies from 2 to 4, influenced by extraction methods and harvest season [1,63,64]. A 4-Xyl*p* to 3-Xyl*p* ratio of 3.0 was found for the 1,3;1,4-β-d-xylan of *P. palmata* (Table 1). Galactose linkages accounted for 12% of all linkages, including minor component of 3,6-anhydro-galactose linkages (Figure 4, Table S1). There has been no report on the detailed structural elucidation of galactan fractions isolated from *P. palmata*, probably due to their low abundance in the species. A modest presence of 4-Glc*p* linkages (5%) was noted (Table 1), which was in good agreement with a previous report of 2–7% cellulose content in the cell wall of *P. palmata* [59]. Moreover, minimal levels of arabinose and mannose linkages were also observed (Figure 3, Table 1).

#### 2.2.2. *Gracilariopsis* sp.

Agarose, an industrially important galactan consisting of alternating 3-Gal*p* and 4-AnGal*p* units, is frequently extracted from the Gracilariaceae family of red seaweeds [65,66]. As expected, the results showed that the AIR of *Gracilariopsis* sp. contained abundant 3-Gal*p* (40%) and 4-AnGal*p* (27%) (Table 1, Figure 7), corresponding to 54% of agarose, assuming all 4-AnGal*p* was attributed to agarose. It is important to note that the ratio of 3-Gal*p* to 4-AnGal*p* was calculated to be 1.5:1, which exceeded the 1:1 ratio of 3-Gal*p* to 4-AnGal*p* in agarose, corresponding to a 12% composition difference between them. This difference closely aligned with the combined compositions (10%) of 4-Gal*p* and its related sulfated forms (2,4-Gal*p*, 3,4-Gal*p*, 4,6-Gal*p*, 2,4,6-Gal*p*, and 2,3,4,6-Gal*p*), suggesting the presence of various minor sulfated galactan components in addition to the major agarose component. Moreover, it could not be ruled out that some of the excess 3-Gal*p* might originate from glycoproteins in this specific species, a hypothesis that requires verification by future studies. The presence of the 4,6-Gal*p* linkage, comprising 3% of the total linkage and being the third most prevalent galactose linkage in the AIR (Table 1, Figure 3), indicated a 4-Gal*p* unit sulfated at the *O*-6 position, potentially acting as a biological precursor to the 4-AnGal*p* linkage [67]. The observation of minor levels of xylose, mannose, and arabinose linkages (Table 1, Figure 3) was in good agreement with the previously reported monosaccharide compositions of this species [65,68,69].

#### 2.2.3. *Prionitis* sp.

*Prionitis* sp., belonging to the family Halymeniaceae and order Halymeniales, is known for producing complex sulfated galactans [1]. The linkage analysis revealed a variety of galactan-related linkages, with 3-Gal*p* (30%) emerging as the predominant linkage, followed by notably high levels of 4-AnGal*p* (12%) and 3,4-Gal*p* (11%), substantial quantities of 2,4-Gal*p* (6%), 4-Gal*p* (5%), 3,6-Gal*p* (5%), 3,4,6-Gal*p* (4%), and 2,3-Gal*p* (4%), and lesser amounts of 4,6-Gal*p* (2%), 2,3,4,6-Gal*p* (2%), 2,4-AnGal*p* (1%), 2,4,6-Gal*p* (1%), and 2,3,6-Gal*p* (1%) (Table 1, Figure 8). Given the diversity of galactose linkages, various galactans with distinct disaccharide units might exist. For instance, 3-Gal*p* could link to 4-AnGal*p* to form the disaccharide unit of β-carrageenan and could link to 4,6-Galp, a biological precursor of 4-AnGal*p*, to form the disaccharide unit of γ-carrageenan. The presence of 3,4-Gal*p* and 4-AnGal*p* suggested the potential existence of the κ-carrageenan disaccharide unit. Additionally, the co-existence of 3,4-Gal*p* and 4,6-Gal*p* suggested the possible presence of a disaccharide unit typical of µ-carrageenan. Moreover, among all the seaweeds analyzed, *Prionitis* sp. contained the highest levels of t-Xyl*p* (2%) and t-Gal*p* (1%), as shown in Table 1, suggesting its AIR sample had more xylosylation and galactosylation than those of the other species.

#### 2.2.4. *Callophyllis* sp.

Among all the red seaweeds examined, *Callophyllis* sp. exhibited the greatest variety of linkages, with no single linkage exceeding the relative composition of 16%, highlighting the complexity of the galactans present in the seaweed. The most abundant galactan-related linkages in *Callophyllis* sp. were 4-AnGal*p* (16%) and 3,6-Gal*p* (15%), followed by a moderate amount of 2,3-Gal*p* (7%), along with many other less abundant linkages (Table 1, Figure 9). Many combinations of monosaccharides could form the disaccharide repeating units of different carrageenans. The abundant 4-AnGal*p* (16%) and 3,6-AnGal*p* (15%) strongly suggested the disaccharide repeating unit of ω-carrageenan [4]. 3-Gal*p* (6%) could link with 4-AnGal*p* (16%) to form the disaccharide unit of β-carrageenan, and with 4,6-Gal*p* (2%) to form the disaccharide unit of γ-carrageenan. 3,4-Gal*p* (4%) could link with 2,4,6-Gal*p* (2%) to form ν-carrageenan, with 2,4-AnGal*p* (2%) to form ι-carrageenan, and with 4,6-Gal*p* (2%) to form µ-carrageenan. Additionally, 2,3-Gal*p* (7%) could link with 2,4,6-Gal*p* (2%) to form the disaccharide unit of λ-carrageenan, and with 2,4-AnGal*p* (2%) to form the disaccharide unit of θ-carrageenan. Notably, a galactan fraction isolated from *Callophyllis hombroniana* was reported to be θ-carrageenan [70]. It was also reported that galactan fractions isolated from *Callophyllis variegata* were highly sulfated [70]. The disaccharide structures of the galactans in this genus need to be further investigated in future studies. Moreover, *Callophyllis* sp. contained a higher level of cellulose than any other seaweed examined, as evidenced by the high 4-Glc*p* composition of 15% (Table 1) and the estimated cellulose composition of 13% (Figure 5, Table S2). The nondetection of terminal linkages t-Xyl*p* and t-Gal*p* (Table 1) supported the fact that the highly acetylated PMAAs were caused by sulfation rather than substitution with xylose and galactose residues, which is in good agreement with previous reports on this genus [71,72].

#### 2.2.5. *Mastocarpus papillatus*

*M. papillatus* is a seaweed belonging to the Phyllophoraceae family within the Gigartinales order. Seaweeds from the Gigartinales order are known to produce carrageenans [71]. Gametophytes from the Phyllophoraceae family are recognized for producing ι- or κ/ι-hybrid carrageenans [73]. The gametophytes of *Mastocarpus stellatus* were reported to have a carrageenan content of 37% (*w*/*w*) dry weight, predominantly from the ‘kappa family’ (κ-, ι-, ν-, μ-carrageenans), with κ- and ι-carrageenans accounting for over 80% of the total carrageenan content [74]. *Mastocarpus pacificus* was reported to contain mainly κ/ι-hybrid carrageenans and low levels of their biological precursors, μ- and ν- carrageenans [75]. As expected, results showed that *M. papillatus* had the highest levels of 3,6-anhydro-galactose linkages among all examined seaweeds, with 4-AnGal*p* and 2,4-AnGal*p* comprising 22% and 12% of the total linkages, respectively, combining to a total AnGal*p* linkage of 34% (Table 1, Figure 10). The linkage 3,4-Gal*p*, with a high composition level of 46%, represented the highest composition value in Table 1. The significant levels of 3,4-Gal*p*, 4-AnGal*p*, and 2,4-AnGal*p* indicated the potential presence of disaccharide unit of 3,4-Gal*p* and 4-AnGal*p* for κ-carrageenan and a disaccharide unit of 3,4-Gal*p* and 2,4-AnGal*p* for ι-carrageenan. The higher levels of 4-AnGal*p* compared to 2,4-AnGal*p* suggested that the gametophyte produces a higher ratio of κ-carrageenan disaccharide unit than ι-carrageenan disaccharide unit. Additionally, some of the extra 3,4-Gal*p* could link to 4,6-Gal*p* (1%) and 2,4,6-Gal*p* (1%) to form the disaccharide units of µ- and ν-carrageenans, serving as the biological precursors of κ- and ι-carrageenans. Moreover, *M. papillatus* demonstrated the lowest cellulose composition among all the seaweeds analyzed (Figure 5, Table S2).

#### 2.2.6. *Mazzaella splendins*

*M. splendens* belongs to the family Gigartinaceae, which is known to synthesize κ- and κ/ι-hybrid carrageenans from gametophytic plants and λ-carrageenans from sporophytic plants [1]. Results showed that the AIR of *M. splendens* contained abundant linkages of 3,4-Gal*p* (20%), 2,3-Gal*p* (18%), 3-Gal*p* (12%), 4-AnGal*p* (10%), 2,4,6-Gal*p* (10%), and 2,4-AnGal*p* (8%) (Table 1, Figure 11). The sum of 4-AnGal*p* (10%) and 2,4-AnGal*p* (8%), totaling 18%, is close to that of 3,4-Gal*p* (20%), and the latter could link with 4-AnGal*p* to form the disaccharide repeating unit of κ-carrageenan and with 2,4-AnGal*p* to produce the disaccharide unit of ι-carrageenan, possibly from the gametophytic phase of the seaweed. Additionally, some extra 3,4-Gal*p* could link with 4,6-Gal*p* (2%) to form µ-carrageenans, the precursor of κ-carrageenan. The presence of λ-carrageenans was supported by the presence of 2,3-Gal*p* (18%) and 2,4,6-Gal*p* (10%), indicating part of the seaweed being in its diploid macroscopic phase. Some extra 2,3-Gal*p* could possibly form ξ-carrageenan with 2,4-Gal*p* (2%) [76]. Moreover, 4-Gal*p* (3%) could potentially link to 4-AnGal*p* to form β-carrageenan. These results were in good agreement with a previous report stating that the carrageenan fractions isolated from a closely related species, *Mazzaella parksii*, showed the presence of various carrageenan structures including κ-, ι-, β-, μ-, ν-, κ/ι-hybrid, and κ/β-hybrid carrageenans [77]. Moreover, the species demonstrated a relatively low 4-Glc*p* composition of 6% (Table 1) and a low estimated cellulose composition of 3% (Figure 5, Table S2).

### 2.3. Chemometric Analysis by PCA

**Figure 12:**
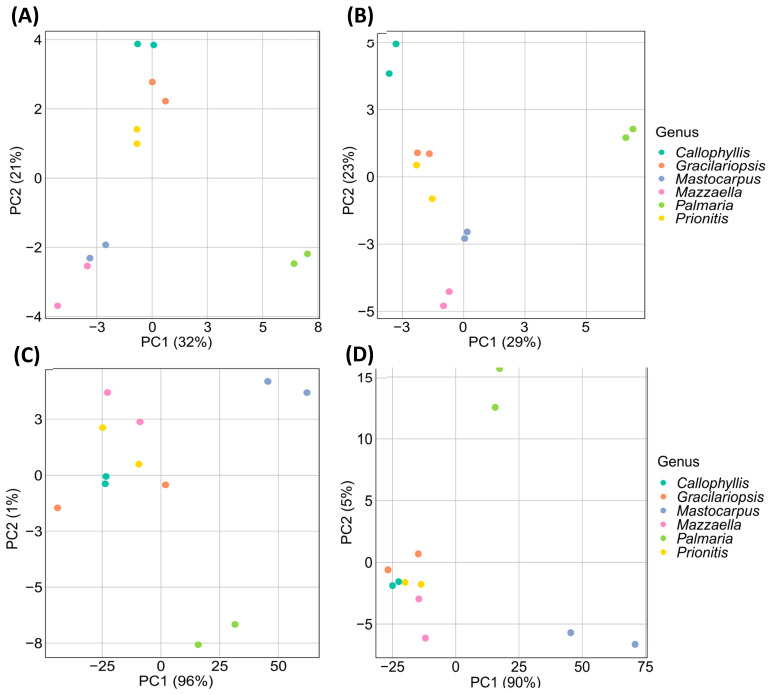
PCA score plots of (**A**) relative linkage compositions calculated from GC-FID measurement, (**B**) relative linkage compositions calculated from GC-MS measurement, (**C**) normalized GC-FID chromatograms of PMAAs, and (**D**) normalized GC-TIC chromatograms of PMAAs. Detailed nominalization procedure for raw GC chromatograms is described in Section 3.5.

Principal component analysis (PCA), the most commonly used chemometric technique [78], aims to reduce data dimensionality, enhance interpretability, and minimize information loss by generating new uncorrelated variables that successively maximize variance [79]. In the current study, PCA was used to analyze four kinds of datasets: relative linkage compositions from GC-MS and GC-FID measurements and normalized GC-TIC and GC-FID chromatograms without any peak assignment, as detailed in Section 3.5. The resulting PCA score plots are depicted in Figure 12.

The PCA score plot from GC-FID-derived relative linkage compositions revealed that the first two principal components (PC1 and PC2) accounted for 29% and 23% (totaling 52%) of the total variances (Figure 12A). The PC1 scores visually separated *P. palmata* from the other five species in the PCA plot, a differentiation likely due to its higher abundance of 3-Xyl*p* and 4-Xyl*p* from 1,3;1,4-β-d-xylan compared to the other analyzed species. Although the other five species could not be well separated based on PC1 scores, the PC2 scores facilitated the visual differentiation of *Callophyllis* sp., *Prionitis* sp., and *Gracilariopsis* sp., while *Mastocarpus papillatus* and *Mazzaella splendens* remained unseparated.

The PCA score plot from GC-MS-derived relative linkage compositions showed that the first two principal components (PC1 and PC2) accounted for 32% and 21% (totaling 54%) of the total variances (Figure 12B). As expected, *P. palmata* demonstrated high positive PC1 scores due to the dominant 1,3;1,4-β-d-xylan linkages, allowing its visual separation from the others. While the other five species could not be well separated based on PC1 scores, they could be partially differentiated based on PC2 scores. The PC2 scores indicated that *Prionitis* sp. and *Gracilariopsis* sp. were closely aligned, with *Callophyllis* sp., *Mastocarpus papillatus*, and *Mazzaella splendens* visually differentiated. Notably, despite *Callophyllis* sp., *Mastocarpus papillatus*, and *Mazzaella splendens* belonging to the same order of Gigartinales, they were well separated from each other due to their different linkage compositions of galactans (Table 1, Figure 3, Figure 9, Figure 10 and Figure 11).

The PCA score plot from the normalized GC-FID chromatograms showed that PC1 accounted for 96% of the total variances (Figure 12C). Spots representing *M. papillatus* and *P. palmata* were visually differentiated from the closely aligned spots of the other four species. *M. papillatus* and *P. palmata* had similar PC1 scores but different PC2 scores, allowing their visual differentiation.

The PCA score plot from the normalized GC-TIC chromatograms revealed that PC1 accounted for 90% of the total variances (Figure 12D). Spots representing *M. papillatus* and *P. palmata* were visually separated from the other three species that were all closely aligned together. *M. papillatus* and *P. palmata* had similar PC1 scores but different PC2 scores, allowing their visual differentiation. Notably, the spots for *M. papillatus* and *P. palmata* appeared in opposite positions compared to their locations on the PCA score plot from the normalized GC-FID chromatograms.

Larger PC1 scores were observed from the PCA score plots of normalized GC-TIC or GC-FID chromatograms than from relative linkage compositions using both detection methods (Figure 12), suggesting that the former methods required fewer principal components to capture the main variances than the latter methods. The normalized chromatograms allowed the visual differentiation of the PCA score spots of M. papillatus from those of the other species. Calculating relative glycosidic linkage compositions from these chromatograms might result in some information loss specific to *M. papillatus*, potentially compromising its visual differentiation from the others by PCA analysis of calculated linkage compositions.

### 2.4. Further Discussions and Future Considerations

This method offers advantages over conventional monosaccharide analysis by enabling the detection of crystalline cellulose along with other polysaccharides in the whole cell walls and extracellular matrix of red seaweeds. The estimated cellulose contents, ranging from 2% to 13%, vary among the 6 red seaweed species (Figure 5, Table S2), indicating the diversity of primary cell wall structures among the species.

In the current study, various linkages of galactose and anhydrogalactose were detected in each of the red seaweed species. The types and abundances of these galactan-related linkages vary among different species (Table 1, Figure 3). However, linkage analysis is fundamentally an analysis of monosaccharide subunits. It provides information about the positions of hydroxyl groups involved in the formation of glycosidic linkages and sulfate substitution. It cannot provide information on the sequence of linkages (e.g., disaccharide structure) because sequence information is lost when polysaccharide chains are depolymerized into monosaccharides during PMAA preparation. We suggest the use of tandem mass spectrometry or nuclear magnetic resonance-based methods to investigate the disaccharide structure of galactans from these species in future studies [20].

Our methodology did not account for naturally occurring methyl groups in red seaweed polysaccharides; information on methyl groups can be determined through ethylation analysis [15] or deuteromethylation analysis [25] instead of normal methylation analysis. The quantitation of pyruvate from algal polysaccharides can be conducted by the releasing and subsequent derivatization into its dinitrophenylhydrazone form [80]. Confirmation of the position of sulfate groups can be achieved by desulfation followed by linkage analysis [1,15,81]. Uronic acid linkages can be determined by reducing the carboxyl group to their corresponding 6,6′-dideuterio neutral sugars before methylation analysis [13]. The current study did not investigate the uronic acid linkages due to their low natural abundance in red seaweed [1].

The quantification of PMAA from glycosidic linkages is based on a relative response factor according to the concept of the effective carbon number of FID or the assumption that the quantities of PMAA molecules are proportional to the ratio of the TIC peak area to the molecular mass of the corresponding PMAA [13,53,82]. There are no purified PMAA standards commercially available that allow for the establishment of a standard calibration curve, as is typically conducted in normal monosaccharide analysis with high-purity monosaccharide standards. Therefore, whole cell wall linkage analysis serves more as an estimation tool and is less accurate compared to conventional monosaccharide analysis [13,82]. Linkage compositions obtained from two separate studies conducted on each AIR sample were very similar to each other, as shown in Figure 3, and with comparable standard deviations to previous reports on plant cell wall linkage analysis [24,27]. These results indicate good repeatability between two separate experiments from the same red seaweed AIR sample. Future studies could examine the consistency of measurements based on larger numbers of samples within and across laboratories to better understand the intermediate precision and inter-lab reproducibility of this method for red seaweeds.

To the best of our knowledge, this is the first report of PCA being applied to analyze the relative linkage compositions and normalized TIC or FID chromatograms. Limited by the number of red seaweed samples, the PCA in this study serves as a preliminary demonstration of its application to linkage analysis data, acting as a proof of concept. Despite such limitations, *M. papillatus* and *P. palmata* can be visually separated from other species in the PCA score plot using normalized TIC or FID chromatograms, without the necessity for peak assignment. The current PCA method primarily serves to portray the proximity of the two data points within each sample, without the application of statistical methods to the data sets from two separate experiments. We suggest that future PCA studies employ statistical approaches to analyze the data sets collected from a large number of seaweed samples.

## 3. Materials and Methods

### 3.1. Materials and Reagents

#### 3.1.1. Seaweed Materials

Dry whole seaweeds of *Gracilariopsis* sp., *Prionitis* sp., *Mastocarpus papillatus*, *Callophyllis* sp., *Mazzaella splendens*, and *Palmaria palmata* were obtained from Canadian Pacifico Seaweeds Ltd. (Surrey, BC, Canada) in October 2020. The dry seaweed was ball-milled into a fine powder using a Retsch Mixer Mill MM 400 ball mill system (Haan, Germany). The dry powders were sealed in 50 mL tubes and stored at −20 °C before analysis.

#### 3.1.2. Chemicals, Reagents, and Consumables

DMSO (Cat. No. 276855), MeI (Cat. No. I8507), NaOH (Cat. No. S5881), methanol (Cat. No. 322415), TFA (Cat. No. 8.08260), glacial acetic acid (Cat. No. 69509), acetic anhydride (Cat. No. 242845), TEAC (Cat. No. 8.21135), 4-MMB (Cat. No. 262323), DCM (Cat. No. 1.00668), sodium bicarbonate (NaHCO_3_) (Cat. No. S8875), 0.5 M methanolic HCl (Cat. No. 07607), Na_2_SO_4_ (Cat. No. 8.22286), acetonitrile (Cat. No. 271004), ethyl acetate (Cat. No. 1.10972), dimethylformamide (Cat. No. 319937), acetone (Cat. No. 179973), barium oxide (BaO) (Cat. No. 288497), and barium hydroxide octahydrate (Ba(OH)_2_·8H_2_O) (Cat. No. 217573) were purchased from Sigma-Aldrich Co., LLC (Burlington, MA, USA). Spectrum Spectra/Por 1 regenerated cellulose (RC) dialysis tubing with molecular weight cut-off (MWCO) of 6000–8000 Da (Cat. No. 08-670D) and NaBD_4_ with an isotopic purity of 99% (Cat. No. 035102.06) were purchased from Thermo Fisher Scientific Inc. (Waltham, MA, USA). Ethanol (Cat. No., P016EAAN) was purchased from Commercial Alcohols Inc. (Chatham, ON, Canada). Deionized water was generated from a Barnstead D0809 Nanopure II system (Thermo Fisher Scientific Inc., MA, USA). All chemical reactions were conducted in Pyrex^®^ disposable glass tubes (thread 15-415, Cat. No. CLS99447161) sealed with Corning^®^ phenolic caps with PTFE liners (Cat. No. CLS999815) and stirred using BRAND^®^ magnetic stirring bars coated with PTFE (8 mm in length and 3 mm in diameter, Cat. No. Z328936), all purchased from Sigma-Aldrich Co., LLC (MA, USA). N_2_ from a cylinder was used to fill the headspace of the tube, and a Stuart evaporator (Cole-Parmer, Vernon Hills, IL, USA) supplied with N_2_ from a generator was used for the evaporation of samples to dryness.

#### 3.1.3. Carbohydrate Standards

Methyl β-d-galactopyranoside (Cat. No. M0285), methyl α-d-glucopyranoside (Cat. No. 66940), methyl α-d-mannopyranoside (Cat. No. 67770), ι-carrageenan (Cat. No. C1138), agarose (Cat. No. A9539), beechwood xylan (Cat. No. X4252,), seaweed carrageenan (Cat. No. C1867), curdlan (Cat. No. C7821), maltose monohydrate (Cat. No. 63418), gum arabic (Cat. No. G9752), gellan gum (Cat. No. G1910), xylan oat spelt (Cat. No. X0627), and laminarin (Cat. No. L9634) were from Sigma-Aldrich Co., LLC (MA, USA). Konjac glucomannan (Cat. No. P-GLCML), wheat arabinoxylan (Cat. No. P-WAXYM), soybean rhamnogalacturonan (Cat. No. P-RHAGN), sugar beet debranched arabinan (Cat. No. P-DBAR), potato galactan (Cat. No. P-GALPOT), ivory nut mannan (Cat. No. P-MANIV), lichenan (Cat. No. P-LICHN), guar galactomannan (Cat. No. P-GGM21), pachyman (Cat. No. P-PACHY), carob galactomannan (Cat. No. P-GALMV), arabinogalactan (Larch Wood) (Cat. No. P-ARGAL), xyloglucan (Cat. No. P-XYGLN), and yeast beta glucan (Cat. No. P-BGYST), arabinoxylan (Cat. No. P-RAXY) were purchased from Megazyme (Wicklow, Ireland). Fucoidan (Cat. No. YF157165), κ-carrageenan (Cat. No. YC30039), λ-carrageenan (Cat. No. YC41782), and psyllium gum (Cat. No. YP58645) were purchased from Biosynth International Inc. (Naperville, IL, USA). α-Lactose (Cat. No. L-204) was from Thermo Fisher Scientific Inc. (Massachusetts, USA). Microcrystalline cellulose was purchased from J.T. Baker (Phillipsburg, NJ, USA).

### 3.2. Preparation of Unfractionated Polysaccharides of Red Seaweeds

AIR samples were prepared according to the literature [23,26], with modifications according to a previous report [83]. Briefly, the sample was soaked in 40 mL of 80% ethanol-deionized water solution (*v*/*v*) three times (8 h, 16 h, then 8 h), with the tube sealed with a PTFE-lined screw cup and rotated on a tube rotator. After each soaking, the sample was centrifuged at 3000× *g* for 30 min, the supernatant was discarded, and the residue was retained for the next round of treatment. The final residue was then subjected to three rounds of washing with acetone (40 mL), followed by three rounds of washing with methanol (40 mL), with the sample being soaked in the solvent for 20 min on the tube rotator for each round. After each wash, the residue was collected by centrifugation at 3000× *g* for 30 min. The residue obtained after the final wash was vacuum-dried using a Savant SPD131DDA SpeedVac concentrator coupled to a Savant RVT5105 refrigerated vapor trap (Thermo Fisher Scientific Inc., MA, USA). The dry pellets were then ball-milled into a fine powder using the aforementioned ball mill system in Section 3.1.1.

### 3.3. Preparation of PMAA Derivatives from Dry AIR Powder

#### 3.3.1. Permethylation

Dry ball-milled fine power of AIR (~10 mg) was suspended in 2 mL of DMSO by magnetically stirring at 60 °C in a glass tube sealed with a PTFE-lined screw cap, with the headspace filled with N_2_. After cooling the tube to room temperature, approximately 200 mg of freshly ground powder from NaOH pellets was added, followed by magnetic stirring the mixture for 2 h in the sealed tube with headspace filled with N_2_. Next, 1.2 mL of MeI was added, and the sample tube was then sealed with a PTFE-lined screw cup and covered with aluminum foil before being subjected to magnetic stirring for 3 h [14,25,26]. The tube containing the reaction mixture was then placed on ice, followed by the addition of 3 mL of DCM, partitioning twice with 5 mL of 10% acetic acid in deionized water (*v*/*v*), then partitioning with deionized water (5 mL) two times. After each partitioning, the upper aqueous phase was carefully collected and pooled into a new tube. Following the final wash, the lower phase was evaporated to dryness under a continuous gentle flow of N_2_. The pooled upper phase was evaporated under N_2_ flow to reduce the volume by half and then was transferred back into the original tube containing the dried lower phase. The resulting mixture was carefully transferred into the RC dialysis tubing with a MWCO of 6000–8000 Da. The original glass tube was washed twice with 1 mL of ethanol and vortexed with cap on after each addition of ethanol. Each ethanol wash was transferred into the same dialysis tubing for dialysis against running water overnight, followed by 24 h of dialysis against 4 L of 0.1 M TEAC, another dialysis for 24 h against 4 L of deionized water, then freeze-drying. The resulting dry samples, in their TEA salt form, were then methylated and cleaned up as previously described, with two exceptions: overnight magnetic stirring in DMSO was conducted at room temperature (instead of at 60 °C), and the dialysis against TEAC was omitted. It was followed by an additional round of overnight magnetic stirring in DMSO at room temperature, permethylation, partitioning, dialysis against running water then deionized water (without TEAC dialysis), and then freeze-drying, as detailed above. A total of three rounds of methylation were conducted.

#### 3.3.2. Hydrolysis and Reduction

The permethylated polysaccharides were redissolved in 8 mL of methanol by magnetically stirring at room temperature in a glass reaction tube. Subsequently, 2 mL of this solution was transferred from the original tube into each of three glass reaction tubes with PTFE-lined screw caps, resulting in a total of four tubes, each containing 2 mL of the methanol solution. After the methanol solution in each tube was evaporated to dryness under N_2_, approximately one-fourth of the total permethylated polysaccharide, corresponding to 2.5 mg of the starting dry AIR, remained dried at the bottom of each tube. Two of these tubes proceeded to the next steps of the derivatization process, while the remaining two were stored at −20 °C, sealed with PTFE-lined screw caps and with the headspace filled with N_2_, for potential future reanalysis or additional experiments.

##### TFA Hydrolysis-NaBD_4_ Reduction

The permethylated polysaccharides dried in the bottom of a glass reaction tube were magnetically stirred with 2 mL of 4 M TFA for 4 h at 100 °C in a glycerol bath, with the tube sealed with a PTFE-lined cap and the headspace filled with N_2_. After that, the tube was cooled to room temperature, followed by evaporation to dryness under a continuous gentle flow of N_2_ provided by an N_2_ generator. To each tube, 2 mL of freshly prepared NaBD_4_ in deionized water (10 mg/mL, *w*/*v*) was added. The tube was capped and magnetically stirred overnight at room temperature [26]. Subsequently, glacial acetic acid was added dropwise until the fizzing caused by the release of H_2_ ceased, before the sample was evaporated to dryness under N_2_ flow.

##### Reductive Hydrolysis

The reductive hydrolysis was conducted according to the literature [19]. To a tube containing the dried methylated polysaccharides, 0.1 mL of freshly prepared 80 mg/mL 4-MMB deionized water solution and 0.4 mL of 3 M TFA were added. The mixture was magnetically stirred at 80 °C for 30 min in a glycerol bath, with the tube sealed by a PTFE-lined cap and the tube headspace filled with N_2_. After cooling the tube to room temperature, an additional 0.1 mL of the 4-MMB solution and 0.6 mL of 2 M TFA were added, followed by gentle magnetic stirring at 120 °C for 2 h, while maintaining an N_2_ headspace. The tube was then cooled to room temperature again, 0.2 mL of the 4-MMB solution was added, and the sample was immediately subjected to evaporation to dryness under a gentle flow of N_2_ at 50 °C. After that, 1 mL of acetonitrile was added to each tube, and the sample was evaporated to dryness under N_2_ without heating.

#### 3.3.3. Peracetylation and Final Cleanup

Peracetylation was performed according to the literature [15,48]. To each tube containing the dry partially methylated alditol sample, 0.25 mL of TFA was added followed by the addition of 1.25 mL of acetic anhydride. The mixture was magnetically stirred at 60 °C for 1 h, with the tube sealed by a PTFE-lined cap and the tube headspace filled with N_2_. After cooling to room temperature, the sample was evaporated to dryness. DCM (3 mL) and a saturated deionized water solution of NaHCO_3_ (3 mL) were added to the tube, followed by vigorous magnetic stirring with the cap loosely attached until the generation of bubbles ceased. After that, the aqueous phase was discarded, and 3 mL of saturated NaHCO_3_ solution was added. The sample was magnetically stirred for 10 min with the cap loosely on, after which the aqueous phase was discarded, 3 mL of deionized water was added, and the tube was sealed with a PTFE-lined screw cap for an additional 20 min of rotation on the tube rotator. The partitioning with deionized water was repeated two more times to ensure the removal of residual NaHCO_3_. The final upper phase was discarded, and the lower DCM phase was filtered through a glass wool-clogged Pasteur pipette loaded with anhydrous Na_2_SO_4_ powder [41]. The filtrate was evaporated to dryness under N_2_ flow, redissolved in ethyl acetate, and then transferred to a GC vial for GC-MS/FID analysis.

For each sample, two separate experiments were conducted for Section 3.3.1, Section 3.3.2 and Section 3.3.3.

### 3.4. Preparation of PMAA Standards

#### 3.4.1. Preparation of PMAA Derivatives from Polysaccharide Standards

Each sulfated polysaccharide standard (~10 mg) was dissolved in 2 mL of deionized water by magnetic stirring at room temperature overnight. The resulting solution was then transferred into dialysis tubing for dialysis against 4 L of 0.1 M TEAC for 24 h, followed by further dialysis with 4 L of deionized water for 24 h, before being freeze-dried. The resulting dry sulfated polysaccharide in its TEA salt form was then dissolved in DMSO by gentle magnetic stirring at room temperature overnight in a sealed glass reaction tube, with the headspace filled with N_2_. This was followed by permethylation, partitioning, pooling of the concentrated upper phases into the dried lower phase, dialysis of the mixture against running water overnight followed by dialysis against 4 L of deionized water for 24 h, and then freeze-drying, as detailed in Section 3.3.1. No additional round of methylation was conducted. Each neutral polysaccharide standard was suspended in DMSO by magnetic stirring at 60 °C overnight, followed by permethylation, as described in Section 3.3.1, except that during partitioning the upper water phases were discarded and the methylated sample was collected by evaporating the lower DCM phase to dryness, with no need for dialysis or any additional round of methylation. RG-I underwent weak methanolysis (0.5 M methanolic HCl, 80 °C, 20 min) followed by NaBD_4_ reduction [84,85,86] prior to the aforementioned methylation analysis of neutral polysaccharides.

All the permethylated samples were then converted to their PMAAs by 4 M TFA hydrolysis, NaBD_4_ reduction, and peracetylation, as described in Section 3.3.2 and Section 3.3.3. For carrageenans and agarose that contain 3,6-anhydro-galactose, an aliquot of the sample was also subjected to reductive hydrolysis, then peracetylation, to generate PMAAs, as described in Section 3.3.2 and Section 3.3.3.

#### 3.4.2. Preparation of PMAA Derivatives from Methyl Glycosides

Preparation of PMAA standards based on methyl glycosides was conducted according to the literature [87], with modifications. Each methyl glycoside (~50 mg) was dissolved in DMF (2 mL) in a sealed glass reaction tube by magnetic stirring. Subsequently, BaO (200 mg), Ba(OH)_2_·8H_2_O (10 mg), and MeI (1 mL) were added to the tube, which was then sealed with a PTFE-lined cap. The tube was covered with aluminum foil to create a dark environment. The mixture was magnetically stirred, and aliquots of 250 μL of the reaction mixture were taken at 15, 30, 45, 60, 90, and 120 min following the start of the reaction. Each aliquot was poured into 5 mL of methanol, followed by evaporation to dryness under a gentle N_2_ flow at room temperature. After that, the sample underwent peracetylation and was then cleaned up by partitioning and filtering through an anhydrous Na_2_SO_4_ column, as described in Section 3.3.3. The purified peracetylated methyl glycoside was then subjected to 4 M TFA hydrolysis, NaBD_4_ reduction, and peracetylation for generating PMAAs, as detailed in Section 3.3, except that the 4 M TFA hydrolysis at 100 °C was conducted for 2 h (instead of 4 h).

### 3.5. GC-MS and GC-FID Analysis of PMAAs

The PMAA derivatives were tested on an Agilent 7890A-5977B GC-MS system (Agilent Technologies, Santa Clara, CA, USA) installed with a medium-polarity Supelco SP-2380 column (60 m × 0.25 mm × 0.2 µm; Sigma-Aldrich, MA, USA) with oven temperature programmed to start at 55 °C, followed by increases at 20 °C/min to 170 °C, at 0.3 °C/min to 190 °C, and then at 3 °C/min to 215 °C (hold 20 min). A solvent delay of 12 min was used. The same sample was also tested on an Agilent 7890A GC-FID system (Agilent Technologies, CA, USA), equipped with another identical Supelco SP-2380 column, using the same oven program as the GC-MS run. Both GC-FID and GC-MS runs had the same settings of an inlet temperature (250 °C) and constant column helium flow of 0.8 mL/min. Splitless injection and split injection (10:1 ratio) were used for the GC-FID and GC-MS, respectively. The ion source temperature was maintained at 230 °C, and an ionization energy of 70 eV was used for EI ionization. The quadrupole temperature was set at 150 °C, and the transfer line temperature was set at 280 °C. The mass spectrometer was operated in full scan mode, covering a mass range of 45–350 *m*/*z*, with a scan speed of 4.59 scans/s. The FID detector temperature was set to 300 °C, with a dry air flow of 400 mL/min, H_2_ flow of 30 mL/min, and a make-up N_2_ flow of 25 mL/min. Data acquisition, peak assignment, and integration were performed through Agilent OpenLab CDS software version 2.5 (Agilent Technologies, CA, USA). Identification of the PMAAs was based on the comparison of retention times and EI-MS spectra of the PMAAs with those of reference derivatives generated from carbohydrate standards, and by referring to the literature [82]. Two methods for quantifying PMAAs were compared: one based on the GC-TIC chromatogram, and the other on the GC-FID chromatogram. Quantitation using the TIC chromatogram involved calculating the relative molar composition by dividing the peak area of each PMAA by its molecular mass, following the published protocol [13]. Quantitative analysis via GC-FID chromatography relied on FID response factors derived from the concept of an effective carbon number, as detailed in a previous study [53], except 3,6-anhydro-galactose linkages. For the 4-AnGal*p* linkage, the FID response factor was determined experimentally to be 0.49, based on methylation analysis using an agarose standard with a 1:1 molar ratio between 4-AnGalp and 3-Galp, the latter having a response factor of 0.74 [53]. From the established response factor for 4-AnGal*p*, the response factor for the 2,4-AnGal*p* linkage was calculated to be 0.54, according to the principle that an acetyl group substitution at the *O*-2 position, in place of a methyl group, results in a 0.05 increase in the relative response factor [53]. Relative polysaccharide compositions were estimated from the relative linkage compositions by assigning glycosidic linkages to corresponding polysaccharide structures and then summing up all the linkage values grouped under each polysaccharide structure [13]. The content of the Floridean starch was estimated based on the relative composition of 4,6-Glc*p*, according to the average degree of branching of 4.8 [88]. The detailed assignment of linkages to relevant polysaccharides is shown in Table S3. All figures related to the linkage analysis results were produced using GraphPad Prism version 8.0.2 (GraphPad Software Inc., La Jolla, CA, USA), except that the visually accessible bubble plot depicting the relative linkage composition was generated using R-Studio (Posit PBC, Boston, MA, USA) with the ggplot2 [89] and reshape2 [90] packages. Complete R codes for making the figures are included in the Supplementary Document.

### 3.6. PCA Analysis

PCA analysis was conducted based on four different types of data: normalized GC-TIC chromatograms of PMAAs, normalized GC-FID chromatograms of PMAAs, relative linkage compositions calculated from GC-MS measurement, and relative linkage compositions calculated from GC-FID measurement. The linkage composition data were already normalized, while the collected raw GC-FID and GC-TIC required normalization before proceeding with PCA analysis. For each raw chromatogram of GC-TIC or GC-FID, signals collected within the range of retention times from 25 to 110 min were normalized in two aspects. First, the absolute retention time (min) for each time point was converted to relative retention time by dividing by the absolute retention time at the highest point of the 4-Glc*p* peak. Similarly, the absolute abundance reading for each time point was converted to relative abundance by dividing by the absolute abundance at the highest point of the 4-Glc*p* peak. Each data file was transferred onto an Excel sheet for PCA analysis using R-Studio packages dplyr [91], forcats [92], and ggplot2 [89], with the script incorporating Z-score standardization to scale the datasets into a standard normal distribution prior to the PCA analysis. Complete R codes for the analysis are included in the Supplementary Document.

## 4. Conclusions

In conclusion, this improved linkage method has proven to be a useful tool for studying polysaccharide structural variations across red seaweed species. The unfractionated polysaccharides of *Palmaria palmata* predominantly comprise mixed-linkage xylans, supplemented by smaller quantities of sulfated galactans and cellulose. In contrast, the polysaccharides of the other five species are rich in sulfated galactans, with a variety of linkages in 3,6-anhydro-galactose and galactose with varied sulfation patterns. This study also noted varying cellulose levels among species, highlighting the superiority of the method in cellulose analysis over the conventional method of GC-based monosaccharide analysis, which typically underestimates glucose in crystalline cellulose. Furthermore, an enhanced response in anhydro sugar linkages was observed from FID detection compared to EI-MS detection. For the first time, the application of PCA to these linkage composition values and normalized GC-TIC and GC-FID chromatograms without peak assignments has been explored, as a proof-of-concept demonstration of the method’s capacity and potential to distinguish various red seaweed species.

## Figures and Tables

**Table 1:** Linkage compositions (Mol%) of unfractionated polysaccharides in red seaweeds determined by GC-FID.

Linkage	*Prionitis*	*Gracilariopsis*	*Callophyllis*	*Mastocarpus*	*Palmaria*	*Mazzaella*
4-AnGal*p*	11.6 ± 0.2	27.1 ± 0.9	15.7 ± 0.0	21.8 ± 0.2	Tr.	9.7 ± 0.8
2,4-AnGal*p*	1.1 ± 0.0	Tr.	2.4 ± 0.2	12.3 ± 0.6	Tr.	8.0 ± 3.5
2-Ara*f*	0.5 ± 0.1	1.1 ± 0.1	2.3 ± 0.1	0.9 ± 0.0	2.0 ± 0.4	Tr.
t-Gal*p*	0.7 ± 0.2	Tr.	Tr.	0.5 ± 0.0	Tr.	Tr.
2-Gal*p*	1.8 ± 0.7	2.7 ± 0.7	1.3 ± 0.2	1.1 ± 0.2	0.7 ± 0.2	1.3 ± 0.2
3-Gal*p*	29.8 ± 1.4	39.5 ± 1.7	5.6 ± 1.2	1.9 ± 0.4	5.6 ± 0.7	11.5 ± 1.6
4-Gal*p*	5.3 ± 0.4	1.9 ± 0.4	5.8 ± 0.7	2.1 ± 0.1	0.9 ± 0.0	3.4 ± 0.1
6-Gal*p*	Tr.	Tr.	Tr.	Tr.	Tr.	Tr.
2,3-Gal*p*	3.5 ± 0.2	Tr.	7.0 ± 0.3	Tr.	Tr.	18.4 ± 2.6
2,4-Gal*p*	6.0 ± 0.3	Tr.	5.6 ± 0.7	Tr.	Tr.	1.5 ± 0.1
2,6-Gal*p*	Tr.	Tr.	Tr.	Tr.	Tr.	Tr.
3,4-Gal*p*	10.8 ± 0.4	2.5 ± 0.1	4.4 ± 0.2	46.2 ± 0.2	1.6 ± 0.1	19.4 ± 1.1
3,6-Gal*p*	4.5 ± 0.1	1.3 ± 0.0	14.6 ± 0.4	1.3 ± 0.0	Tr.	1.7 ± 0.1
4,6-Gal*p*	1.8 ± 0.6	3.3 ± 0.5	1.8 ± 0.1	1.3 ± 0.1	0.7 ± 0.1	1.5 ± 0.5
2,3,6-Gal*p*	0.5 ± 0.0	Tr.	1.6 ± 0.2	0.6 ± 0.3	Tr.	1.8 ± 0.3
2,4,6-Gal*p*	0.6 ± 0.0	Tr.	2.2 ± 0.1	1.0 ± 0.2	Tr.	9.9 ± 3.7
3,4,6-Gal*p*	4.3 ± 0.3	2.2 ± 0.2	2.0 ± 0.1	2.0 ± 0.1	1.4 ± 0.0	1.5 ± 0.1
2,3,4,6-Gal*p*	1.5 ± 0.0	Tr.	1.6 ± 0.3	Tr.	Tr.	Tr.
t-Glc*p*	Tr.	Tr.	Tr.	Tr.	Tr.	Tr.
3-Glc*p*	Tr.	Tr.	Tr.	Tr.	1.6 ± 0.6	Tr.
4-Glc*p*	9.9 ± 3.5	13.4 ± 2.9	14.9 ± 1.3	2.9 ± 0.3	4.6 ± 0.1	5.7 ± 0.8
3,4-Glc*p*	Tr.	Tr.	Tr.	0.9 ± 0.1	Tr.	0.5 ± 0.3
4,6-Glc*p*	Tr.	0.9 ± 0.3	0.6 ± 0.1	Tr.	Tr.	0.7 ± 0.1
3,4,6-Glc*p*	Tr.	Tr.	Tr.	Tr.	Tr.	Tr.
2,3,4,6-Glc*p*	Tr.	Tr.	Tr.	Tr.	Tr.	Tr.
t-Man*p*	Tr.	Tr.	Tr.	Tr.	Tr.	Tr.
3-Man*p*	Tr.	Tr.	1.3 ± 0.1	Tr.	Tr.	Tr.
4-Man*p*	Tr.	Tr.	1.3 ± 0.0	Tr.	Tr.	Tr.
2,3,6-Man*p*	Tr.	Tr.	Tr.	Tr.	Tr.	Tr.
2,4,6-Man*p*	Tr.	Tr.	Tr.	Tr.	Tr.	Tr.
2,3,4,6-Man*p*	Tr.	Tr.	Tr.	Tr.	Tr.	Tr.
t-Xyl*p*	1.8 ± 0.4	Tr.	0.8 ± 0.0	0.7 ± 0.0	0.6 ± 0.2	1.0 ± 0.0
2-Xyl*p*	Tr.	Tr.	Tr.	Tr.	4.9 ± 0.1	Tr.
4-Xyl*p*	1.1 ± 0.6	1.2 ± 0.2	3.6 ± 0.1	Tr.	54.4 ± 1.9	Tr.
3-Xyl*p*	Tr.	Tr.	0.9 ± 0.0	Tr.	18.4 ± 0.9	Tr.
2,4-Xyl*p*	Tr.	0.5 ± 0.1	Tr.	Tr.	0.6 ± 0.1	Tr.
3,4-Xyl*p*	Tr.	Tr.	Tr.	Tr.	0.5 ± 0.0	Tr.
2,3,4-Xyl*p*	Tr.	Tr.	Tr.	Tr.	Tr.	Tr.

Note: Tr. means trace (Mol% < 0.5%). The mean and standard deviation were calculated based on two separate experiments conducted on each sample.

## Data Availability

The data that support the findings of this study are available upon request.

## Supplementary Materials

The supporting information can be downloaded at https://www.mdpi.com/article/10.3390/md22050192/s1. The supporting documents include supplemental tables for linkage analysis (Tables S1–S3), supplemental GC-TIC and GC-FID chromatograms of PMAAs from carbohydrate standards (Figures S1–S11), supplemental EI-MS spectra and ion fragmentation patterns (Figures S12–S15), supplemental bubble plot of GC-MS-derived linkage compositions (Figure S16), and R Script codes for bubble plot and PCA plot.
