# Supplementary material for: Methylation-GC-MS/FID-Based Glycosidic Linkage Analysis of Unfractionated Polysaccharides in Red Seaweeds
Source: Mar Drugs. 2024 Apr 24;22(5):192. doi: 10.3390/md22050192 (PMC11122361; doi:10.3390/md22050192)
Supplement: Supplementary file 1 [file marinedrugs-22-00192-s001.zip › marinedrugs-2965801-supplementary.pdf]

Supporting Documents

Table of contents

**Contents**	**Page**
Supplemental tables for linkage analysis (**Table S1-S3**)	2
Supplemental GC-TIC and GC-FID chromatograms of PMAAs from carbohydrate standards (**Figure S1-S11**)	3-13
Supplemental EI-MS spectra and ion fragmentation patterns (**Figure S12-S15**)	14-17
Supplemental bubble plot of GC-MS-derived linkage compositions (**Figure S16**)	18
R Script code for bubble plot	19
R Script code for PCR plot	20-21

Table S1. Relative monosaccharide compositions (Mol%) of unfractionated polysaccharides of six red seaweed species quantified by GC-FID

Monosaccharide	*Prionitis*	*Gracilariopsis*	*Callophyllis*	*Mastocarpus*	*Palmaria*	*Mazzaella*
AnGal	12.7±0.2	27.3±0.9	18.1±0.2	34.1±0.6	0.2±0.0	17.7±4.3
Ara	0.5±0.1	1.1±0.1	2.3±0.1	0.9±0.0	2.0±0.4	0.3±0.0
Gal	71.6±3.0	54.1±2.0	54.1±1.8	59.0±0.1	11.6±0.8	72.7±4.8
Glc	10.9±3.7	14.9±3.4	16.3±1.5	4.4±0.5	6.6±0.4	7.3±0.3
Man	0.3±0.1	0.5±0.0	2.9±0.2	0.3±0.0	0.1±0.0	0.3±0.0
Xyl	3.9±0.6	2.1±0.4	6.2±0.2	1.3±0.0	79.5±1.5	1.7±0.2

Note: AnGal, 3,6-anhydrogalactose; Ara, arabinose; Gal, galactose; Glc, glucose; Man, mannose; Xyl, xylose. Two separate experiments were conducted to generate averages and standard deviations.

Table S2. Estimated polysaccharide compositions (Mol%) from linkage compositions of six red seaweed species quantified GC-FID

Polysaccharide	*Prionitis*	*Gracilariopsis*	*Callophyllis*	*Mastocarpus*	*Palmaria*	*Mazzaella*
XN	1.5±0.9	1.2±0.2	4.5±0.2	0.4±0.0	72.8±1.0	0.3±0.0
MN	0.1±0.0	0.4±0.0	1.3±0.0	0.2±0.0	0.0±0.0	0.1±0.0
GN	84.3±3.2	81.4±2.9	72.3±1.6	93.1±0.5	11.7±0.8	90.4±0.5
CE	8.5±3.1	10.2±1.8	12.7±0.9	1.8±0.2	4.0±0.2	3.3±1.1
FS	2.0±0.4	4.4±1.4	3.0±0.5	1.5±0.1	0.9±0.4	3.1±0.3
UA	3.6±0.5	2.4±0.2	6.2±0.0	3.0±0.1	10.7±0.4	2.7±0.3

Note: XN: xylans; MN: mannans; GN: galactans; CE: cellulose; FS: Floridean starch; UA: unassigned linkages. Two separate experiments were conducted to generate averages and standard deviations.

Table S3. Estimation of polysaccharide compositions based on linkage compositions

Polysaccharide	Calculation
XN	Sum of 3-Xyl*p* and 4-Xyl*p*
MN	4-Man*p*
GN	Sum of all Gal*p* and AnGal*p* linkages
FS	Calculated based on 4,6-Glc*p* using an average degree of branching of 4.8
CE	Total 4-Glc*p* subtracted by 4-Glc*p* in FS
UA	All remaining linkages

Note: XN: xylan; MN: mannan; GN: galactan; CE: cellulose; FS: Floridean starch; UA: unassigned linkages.

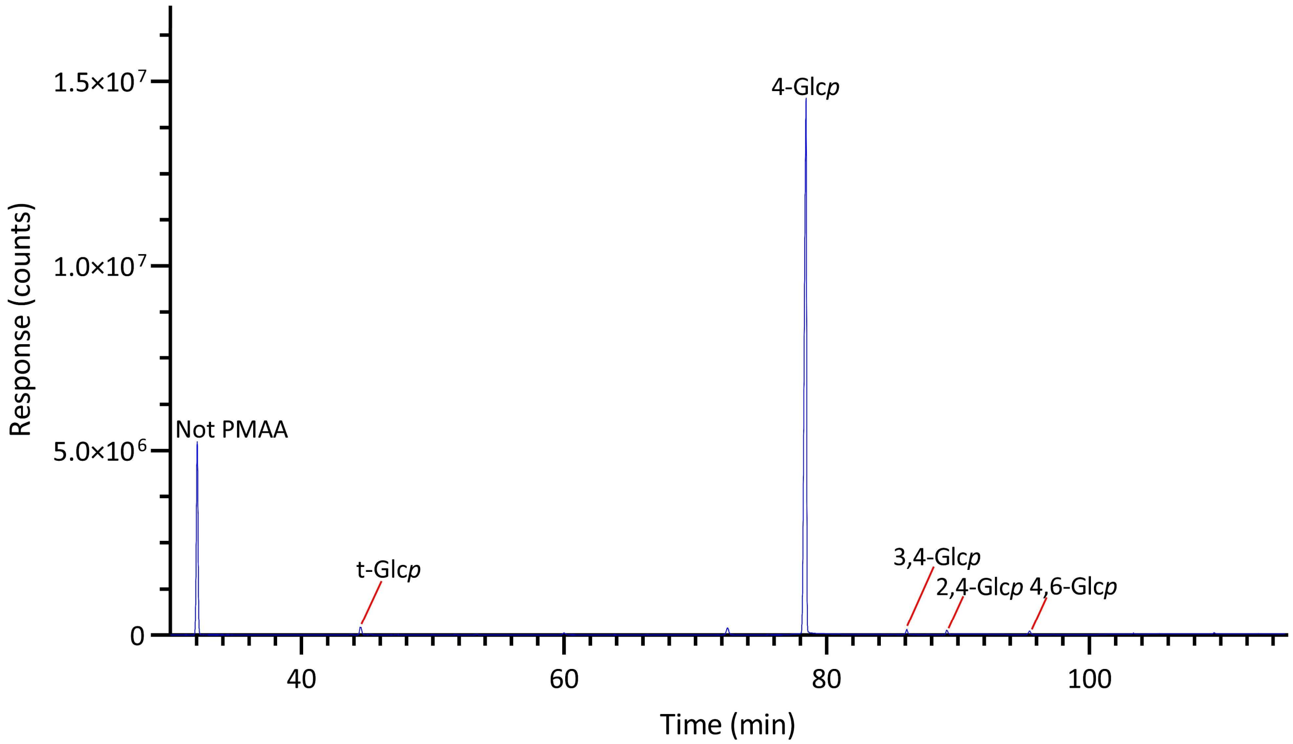

Figure S1. GC-TIC chromatogram of PMAA standards prepared from microcrystalline cellulose.

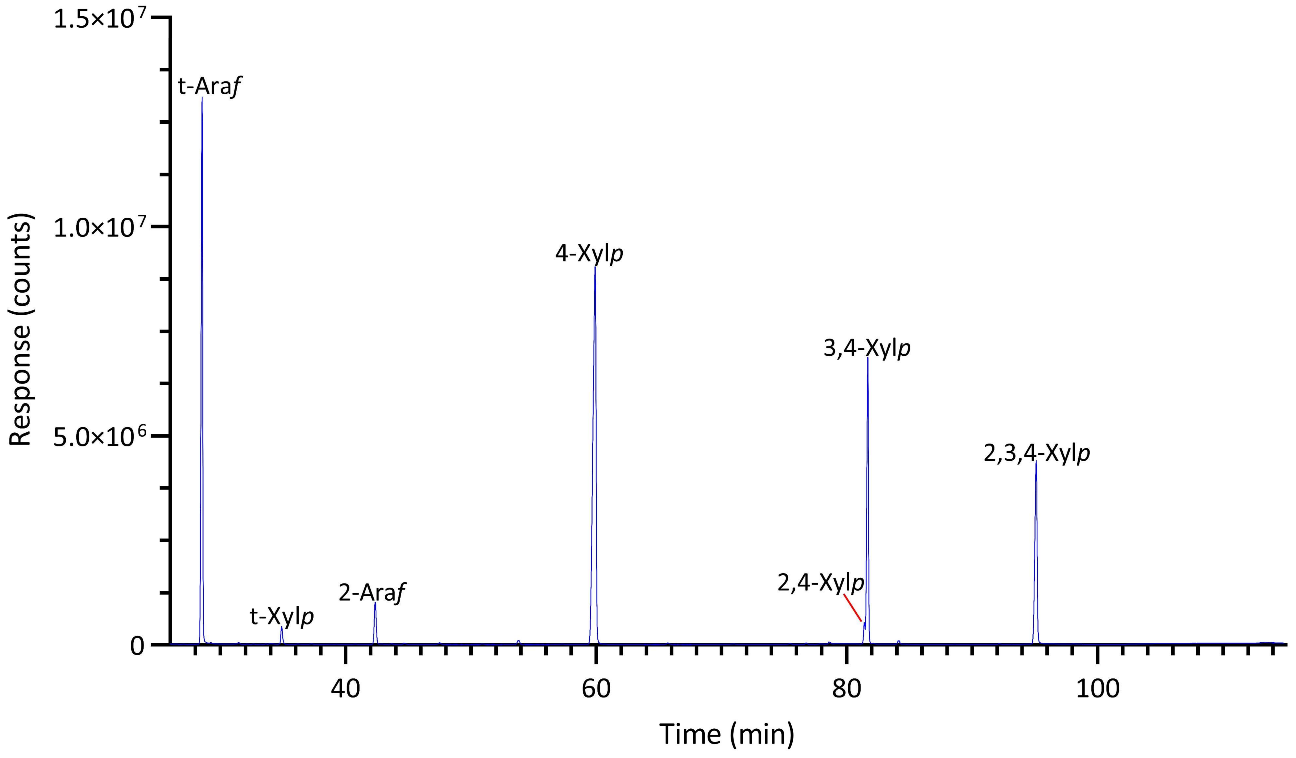

Figure S2. GC-TIC chromatogram of PMAA standards prepared from commercial arabinoxylan.

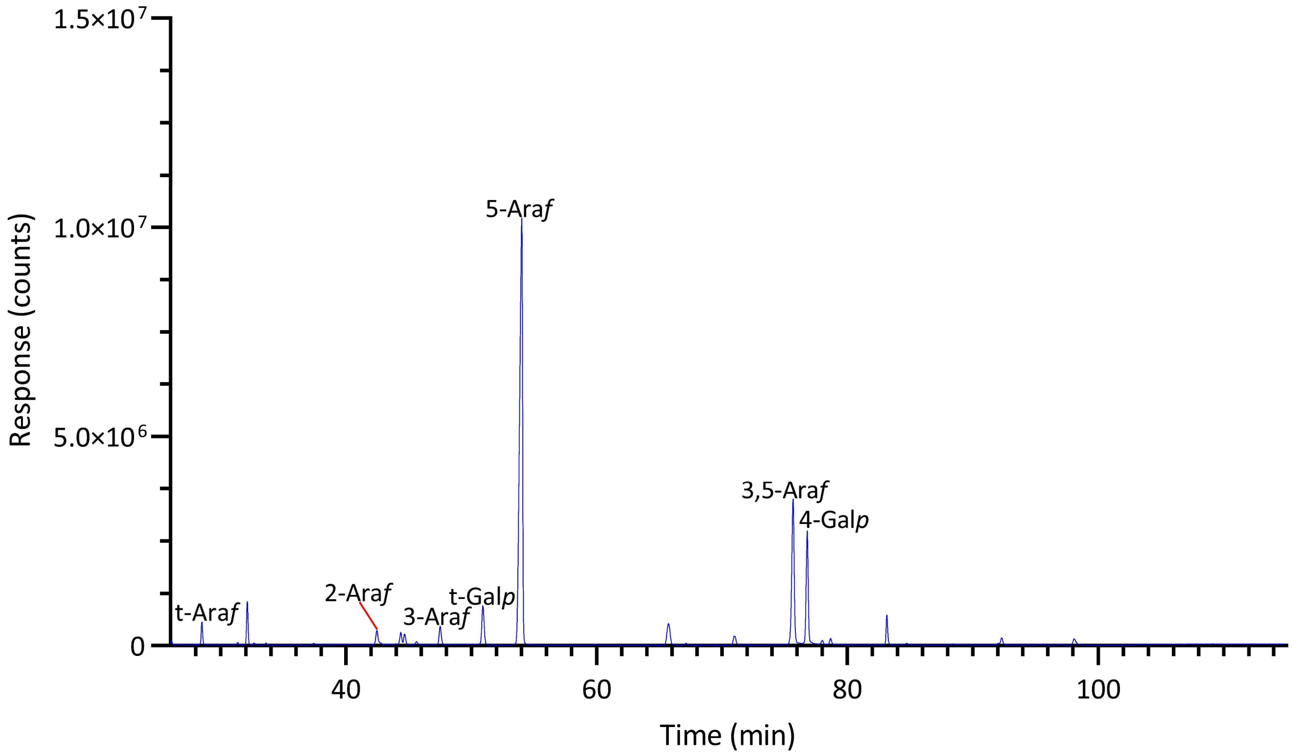

Figure S3. GC-TIC chromatogram of PMAA standards prepared from commercial debranched arabinan.

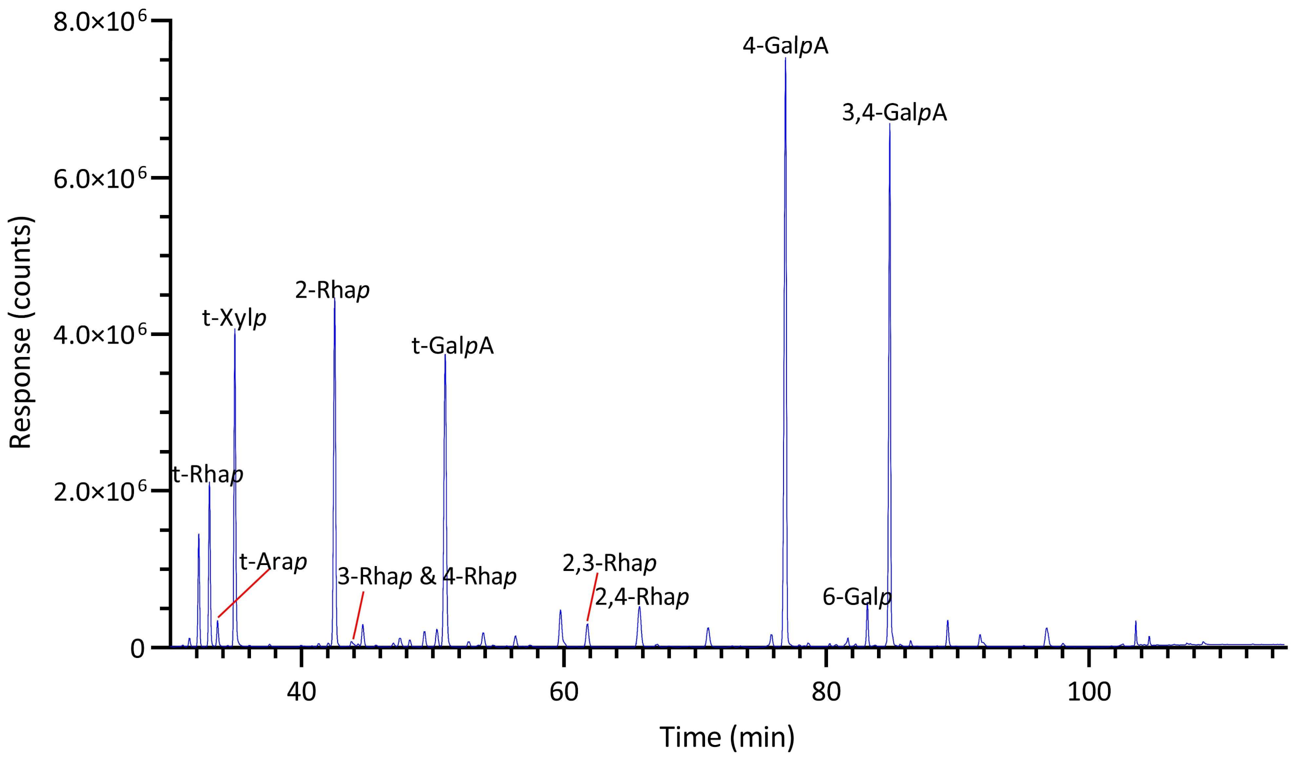

Figure S4. GC-TIC chromatogram of PMAA standards prepared from commercial rhamnogalacturonan I (RG-I)

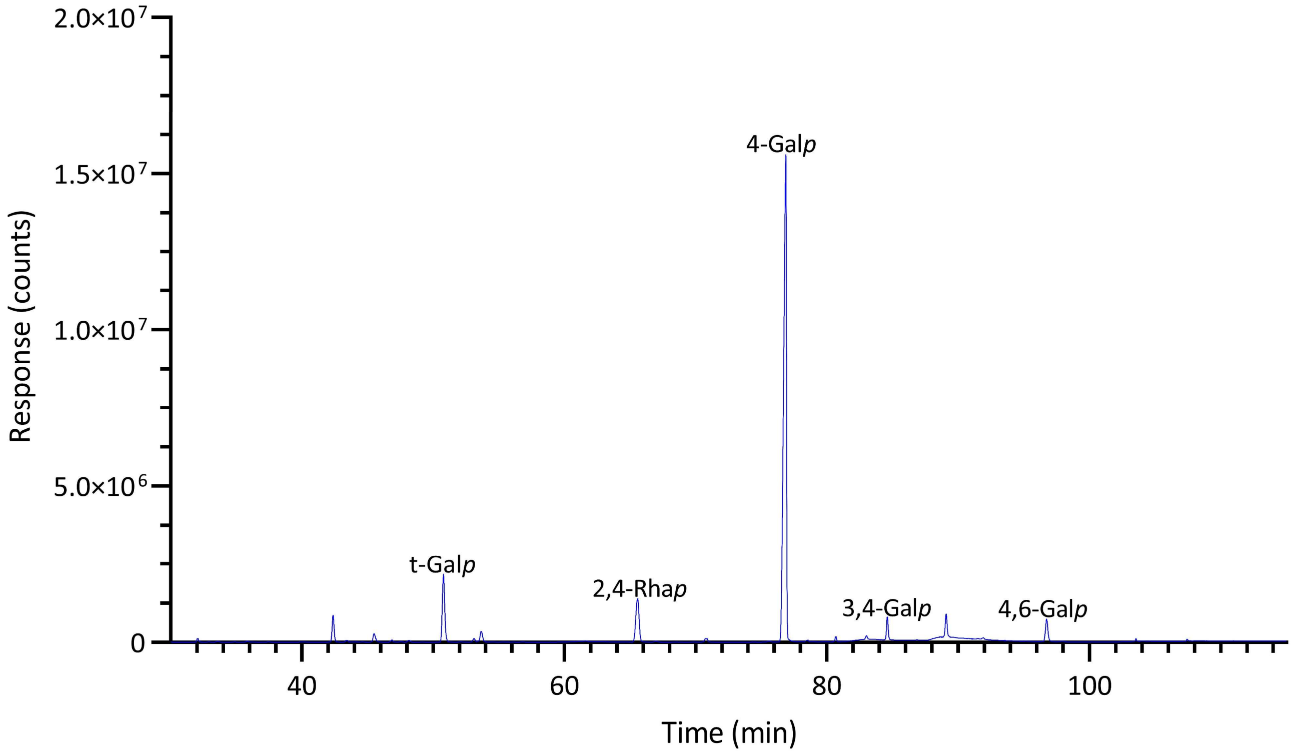

Figure S5. GC-TIC chromatogram of PMAA standards prepared from commercial potato galactan.

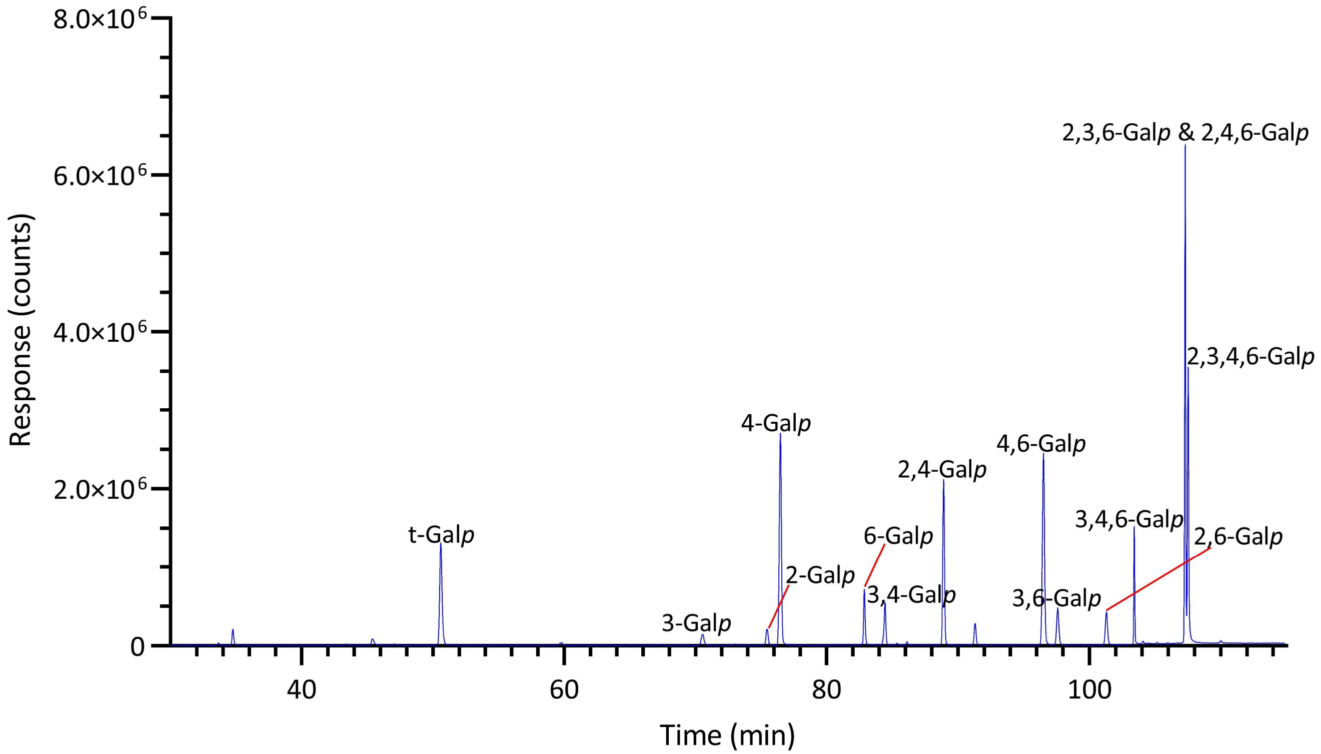

Figure S6. GC-TIC chromatogram of PMAA standards prepared by undermethylation of commercial 1-methyl galactopyranoside.

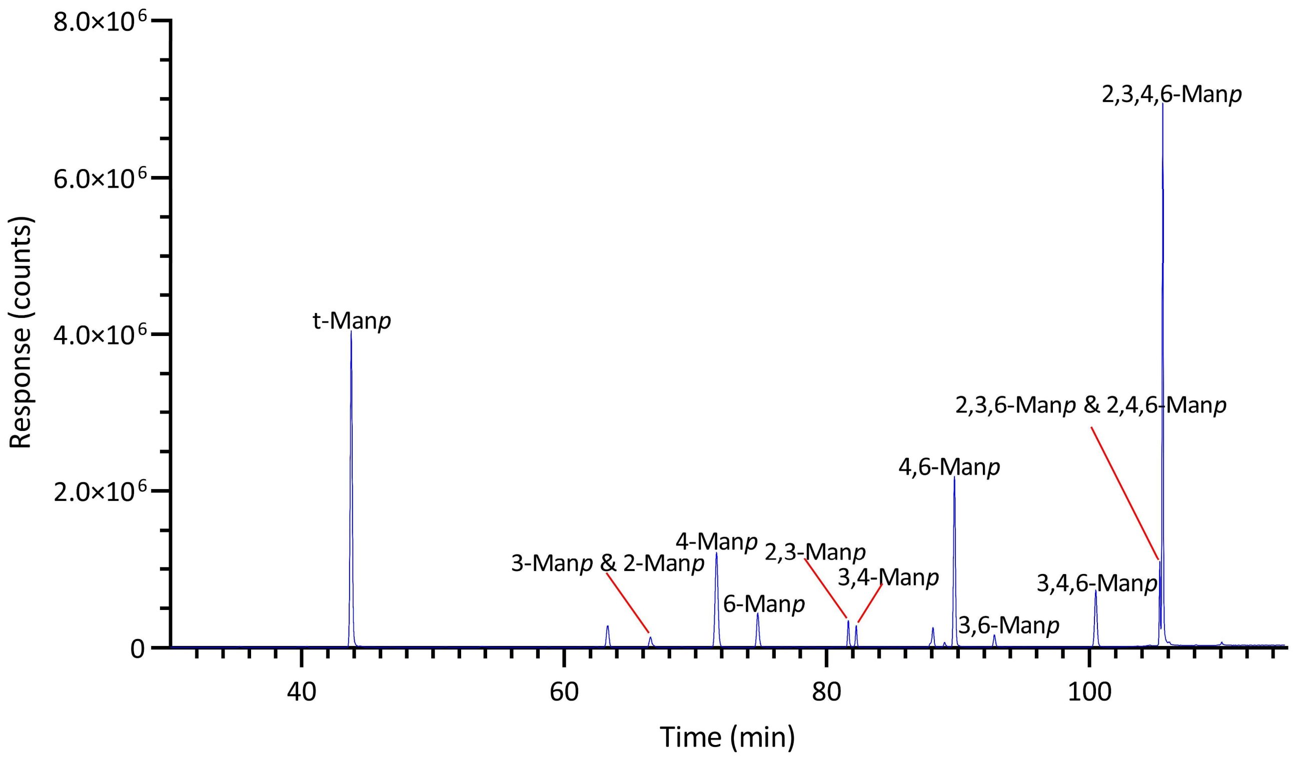

Figure S7. GC-TIC chromatogram of PMAA standards prepared by undermethylation of commercial 1-methyl mannopyranoside.

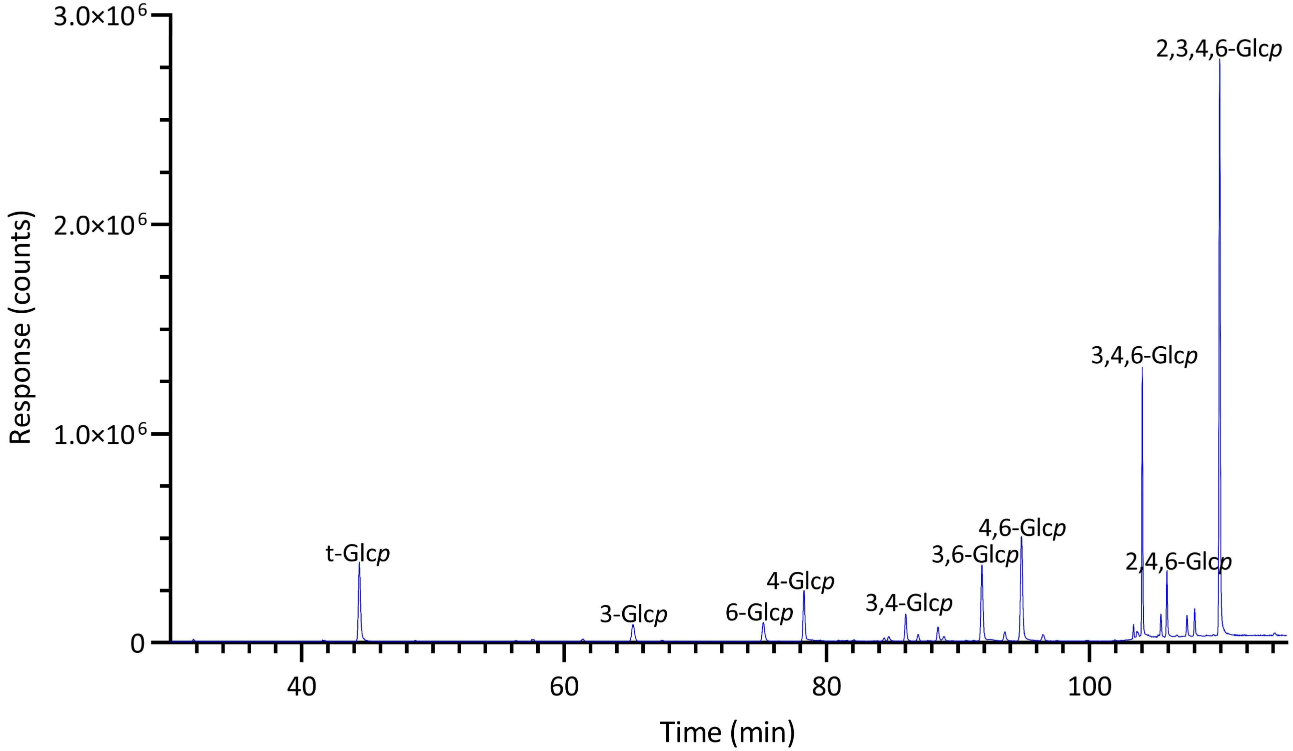

Figure S8. GC-TIC chromatogram of PMAA standards prepared by undermethylation of commercial 1-methyl glucopyranoside.

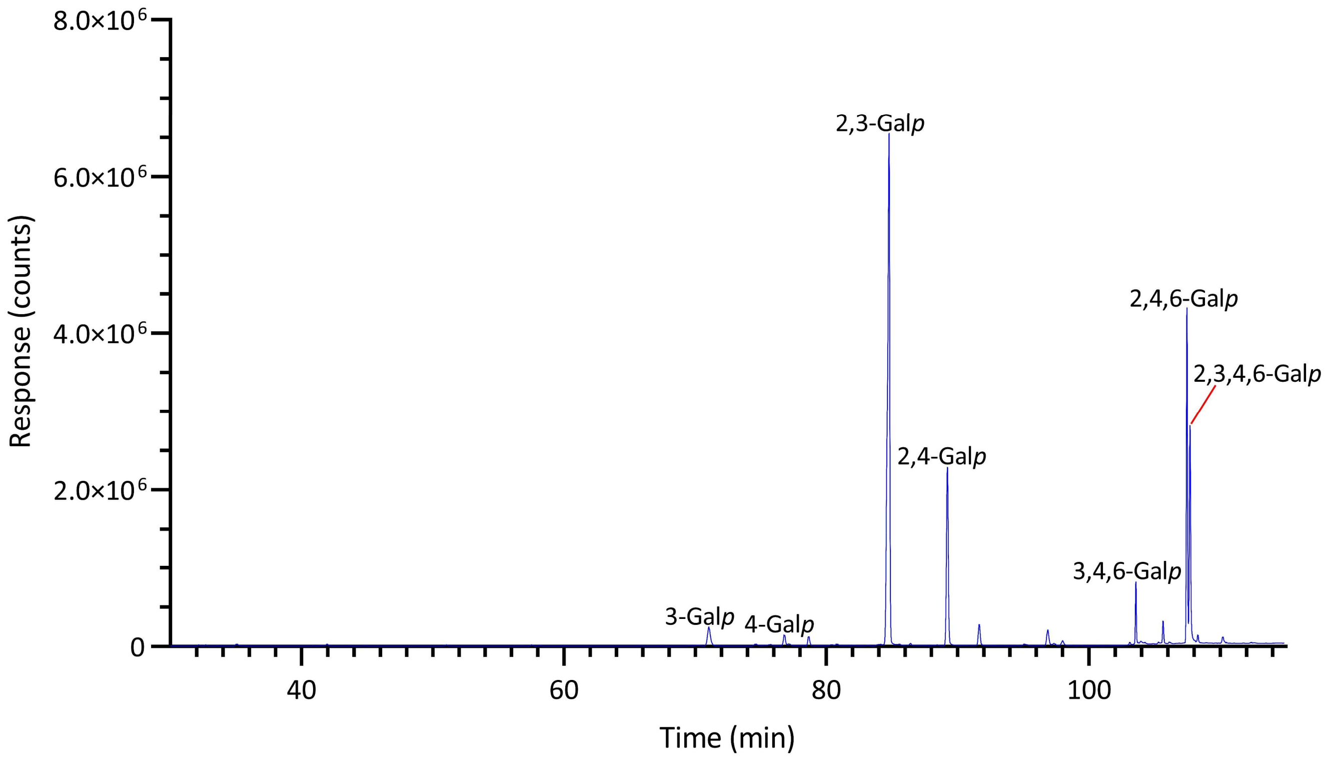

Figure S9. GC-TIC chromatogram of PMAA standards prepared from commercial λ-carrageenan undermethylated by one round of methylation.

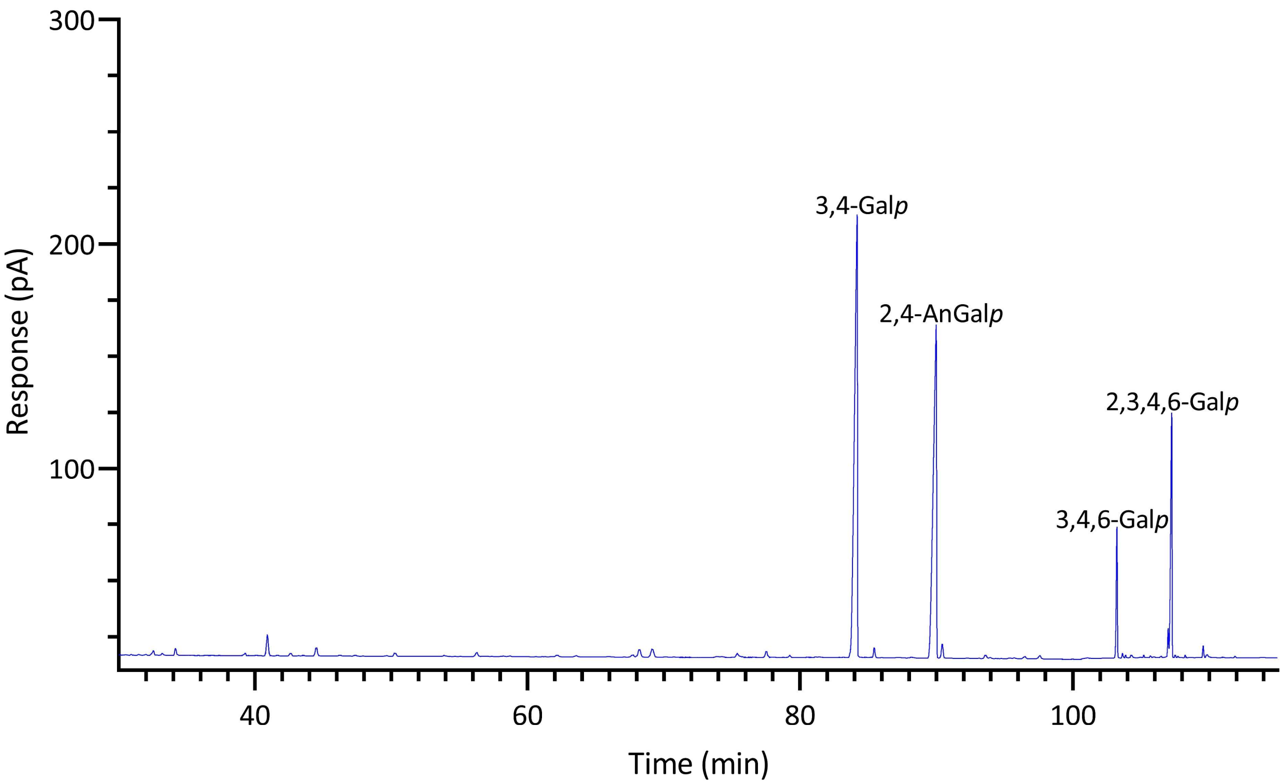

Figure S10. GC-FID chromatogram of PMAA standards prepared from commercial ι-carrageenan undermethylated by one round of methylation.

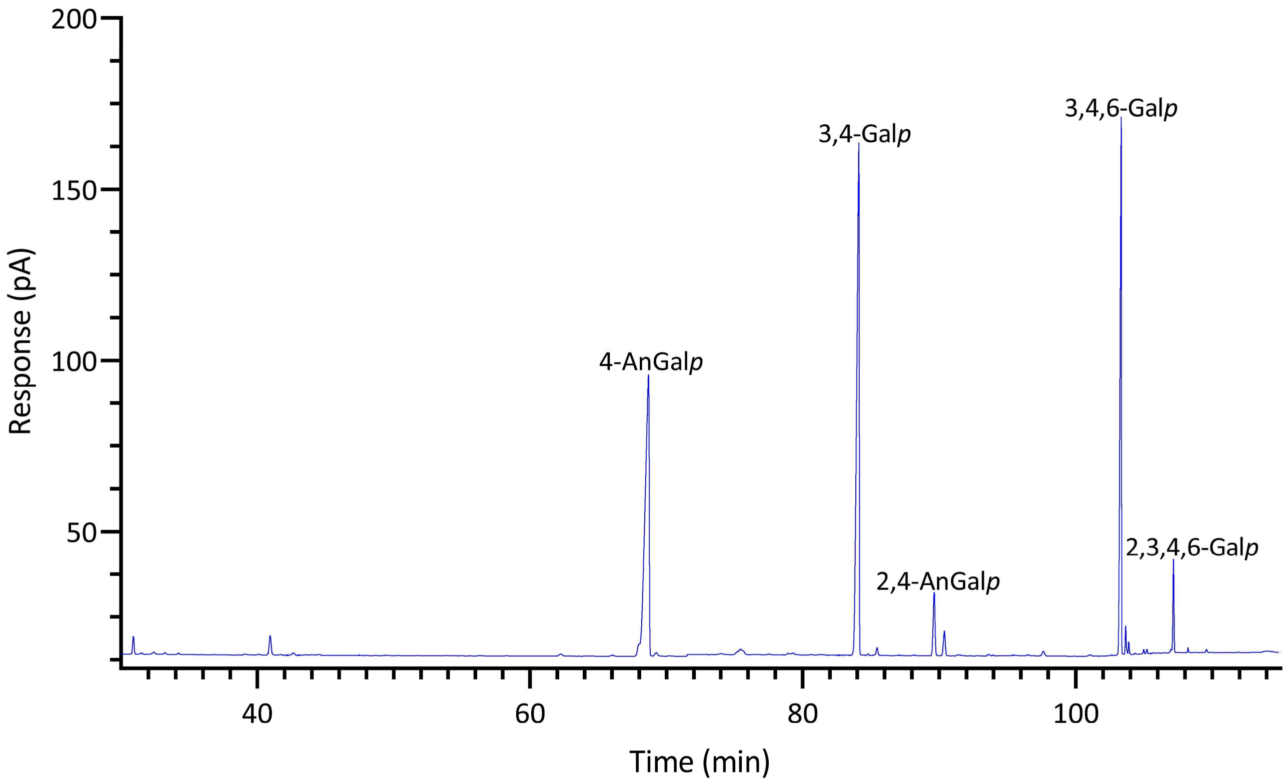

Figure S11. GC-FID chromatogram of PMAA standards prepared from commercial κ-carrageenan undermethylated by one round of methylation.

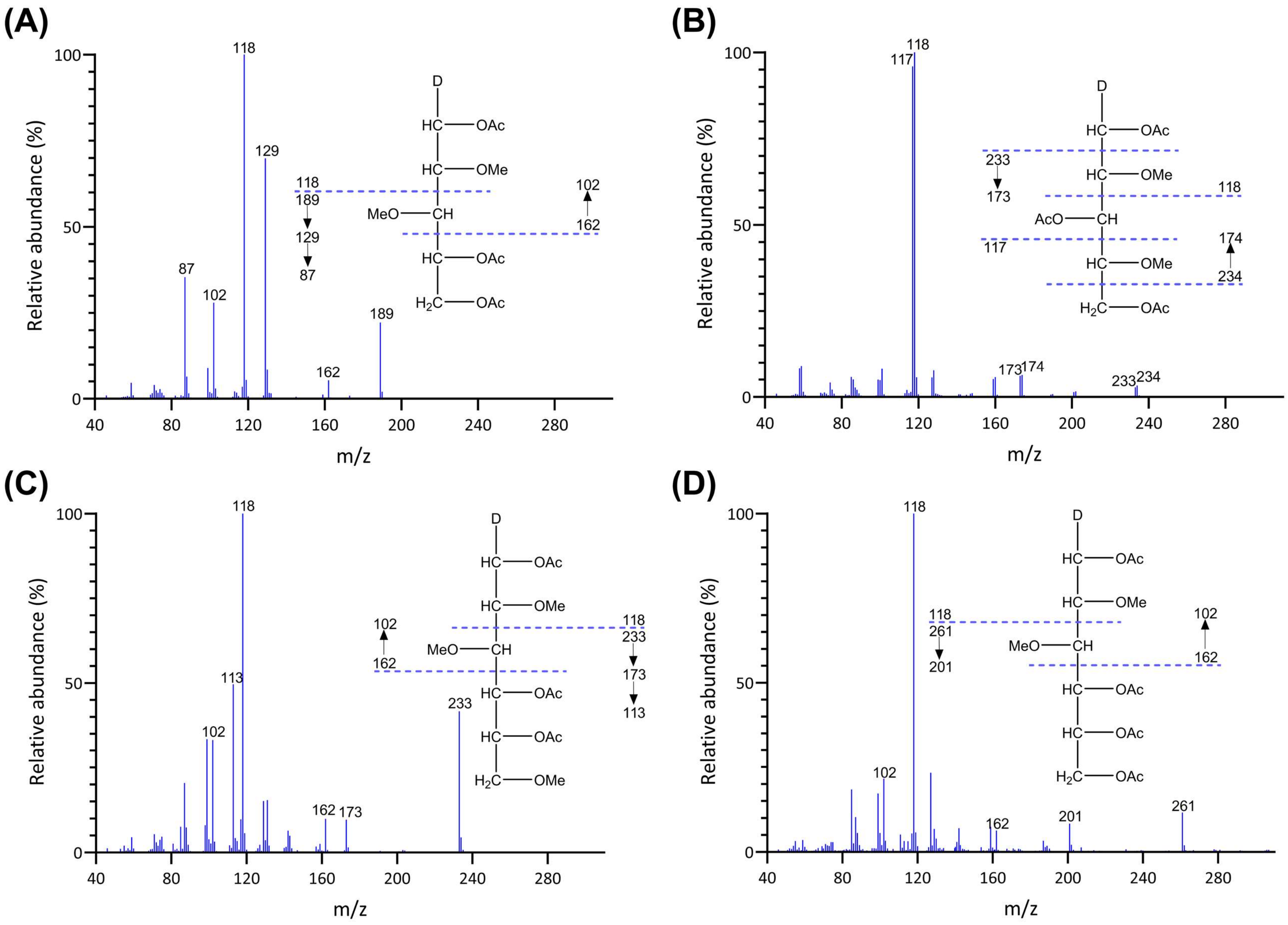
(A)
(B)
(C)
(D)
Relative abundance (%)
m/z
HC—OAc
HC—OMe
MeO—CH
AcO—CH
HC—OAc
HC—OMe
H2C—OAc
H2C—OMe

Figure S12. EI-MS spectra and ion fragmentation patterns of PMAAs from **(A)** 4-Xyl*p*, **(B)** 3-Xyl*p*, **(C)** 4-Glc*p*, and **(D)** 4,6-Glc*p* in *Palmaria palmata*.

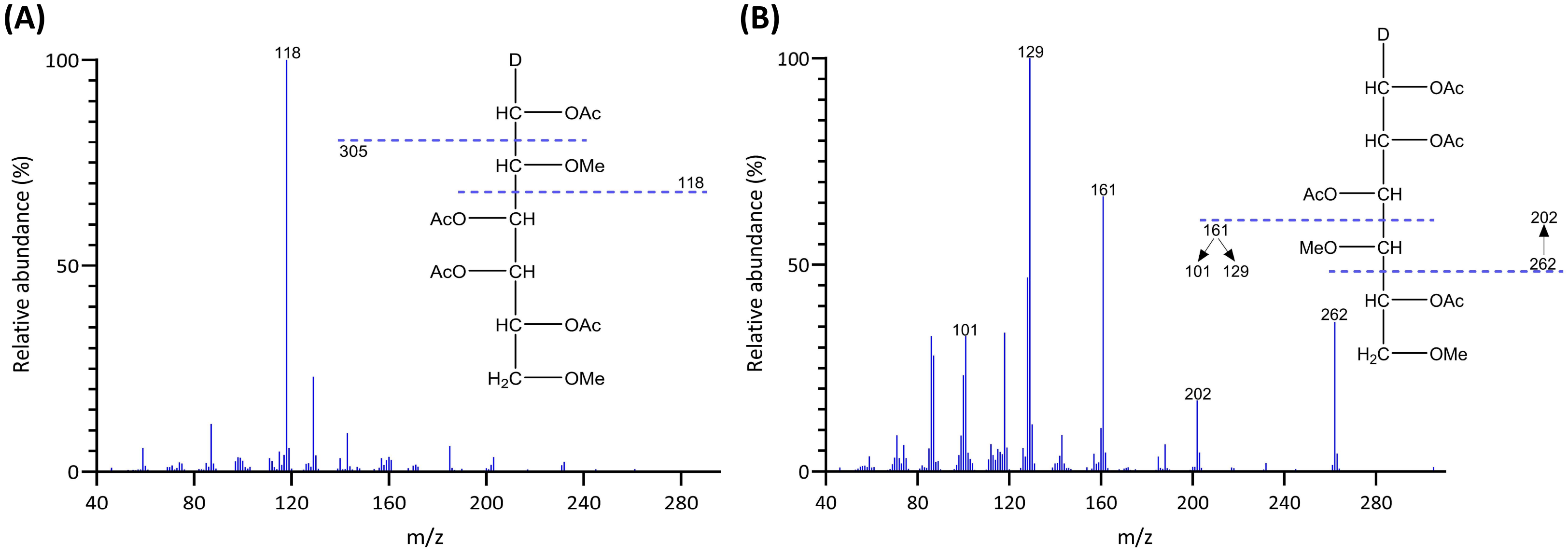

Figure S13. EI-MS spectra and ion fragmentation patterns of PMAAs from **(A)** 3,4-Gal*p* and **(B)** 2,3-Gal*p*.

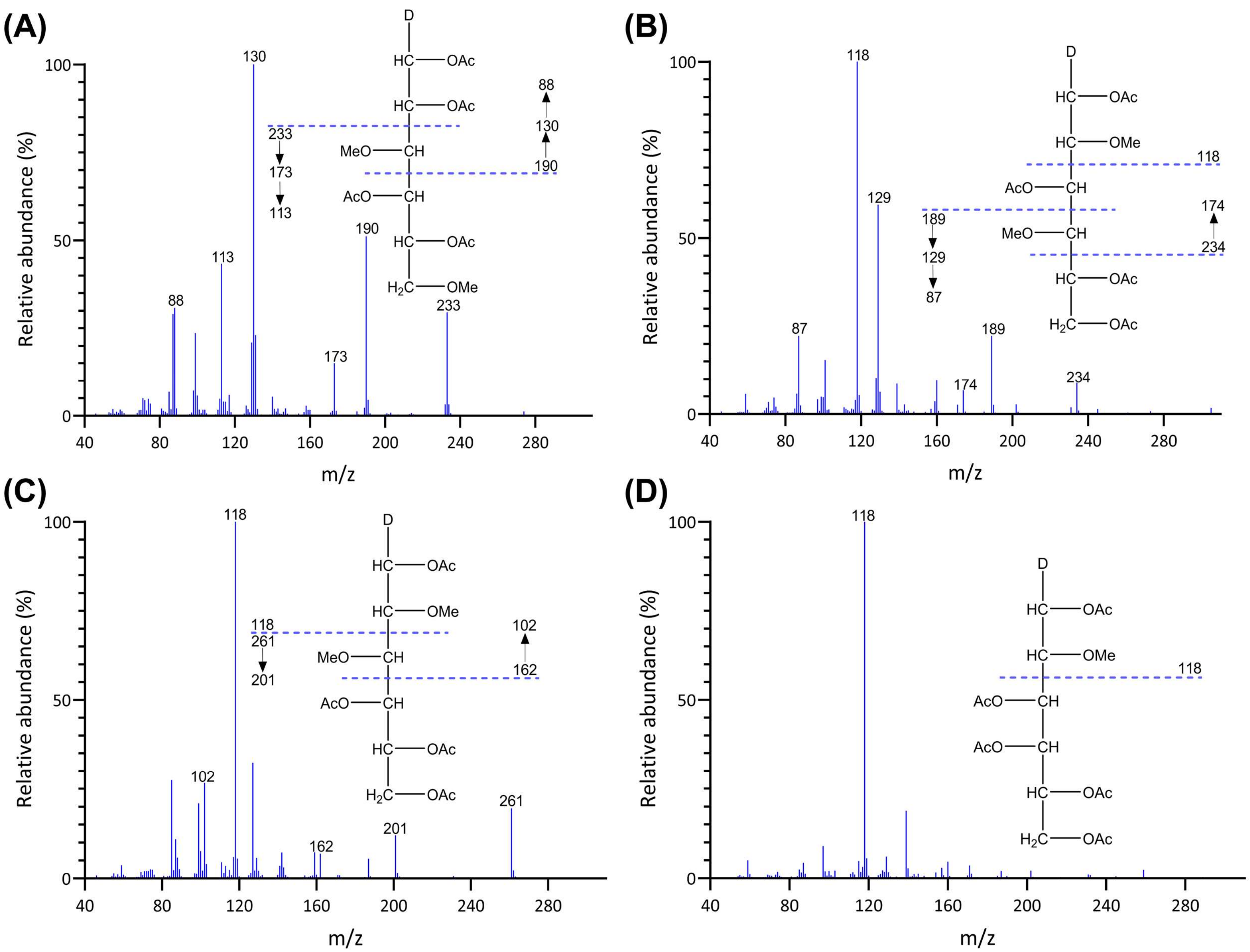
(A)
Relative abundance (%)
m/z
130
113
88
190
173
233
D
HC—OAc
HC—OAc
MeO—CH
AcO—CH
HC—OAc
H_2C—OMe
233 → 173 → 113
190 → 130 → 88
(B)
Relative abundance (%)
m/z
118
129
87
189
174
234
D
HC—OAc
HC—OMe
AcO—CH
MeO—CH
HC—OAc
H_2C—OAc
118
189 → 129 → 87
234 → 174
(C)
Relative abundance (%)
m/z
118
102
162
201
261
D
HC—OAc
HC—OMe
MeO—CH
AcO—CH
HC—OAc
H_2C—OAc
118
261 → 201
162 → 102
(D)
Relative abundance (%)
m/z
118
D
HC—OAc
HC—OMe
AcO—CH
AcO—CH
HC—OAc
H_2C—OAc
118

Figure S14. EI-MS spectra and ion fragmentation patterns of PMAAs from **(A)** 2,4-Gal*p*, **(B)** 3,6-Gal*p*, **(C)** 4,6-Gal*p*, and **(D)** 3,4,6-Gal*p* in *Callophyllis* sp.

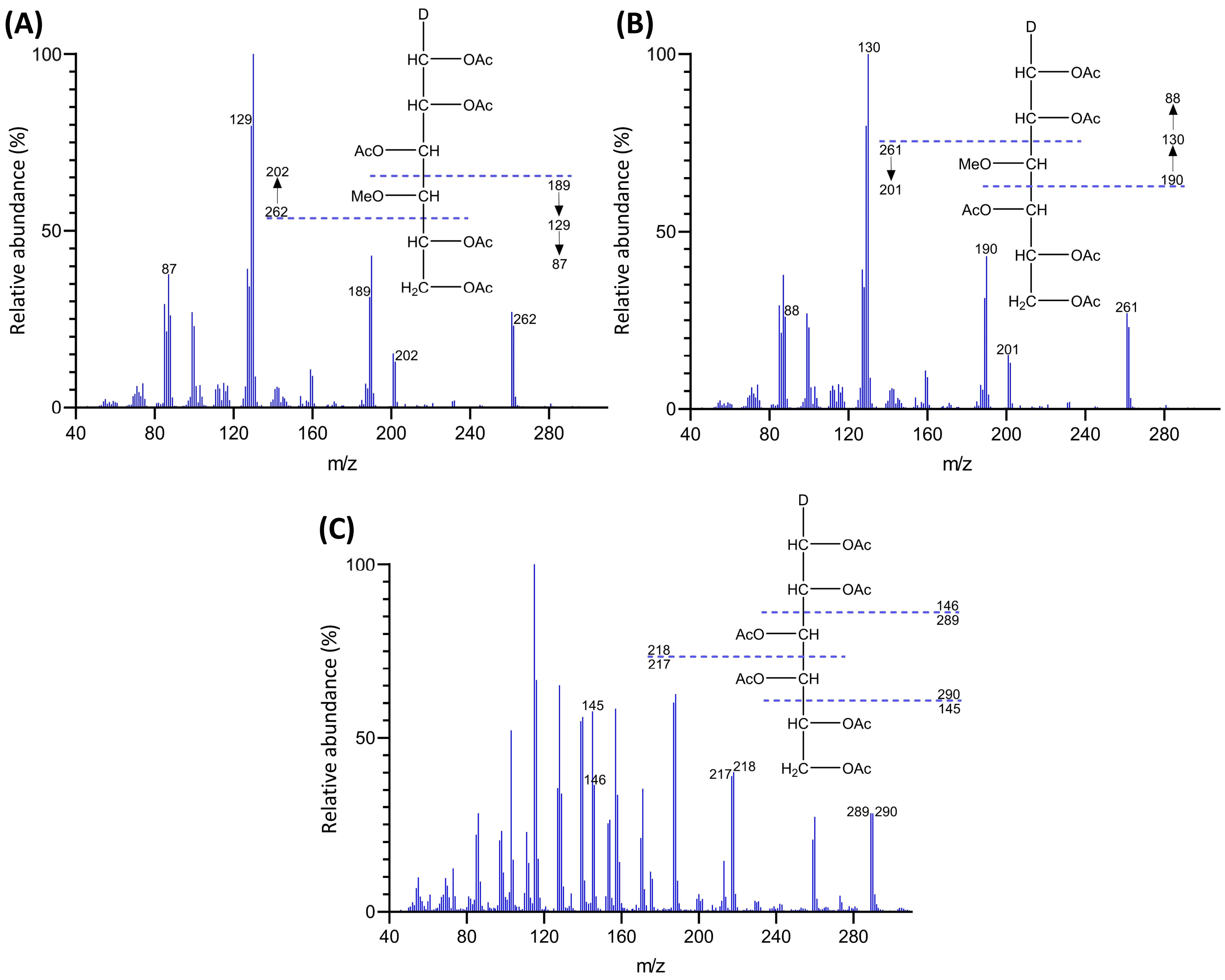

Figure S15. EI-MS spectra and ion fragmentation patterns of PMAAs from **(A)** 2,3,6-Gal*p*, **(B)** 2,4,6-Gal*p*, and **(C)** 2,3,4,6-Gal*p* in *Callophyllis* sp.

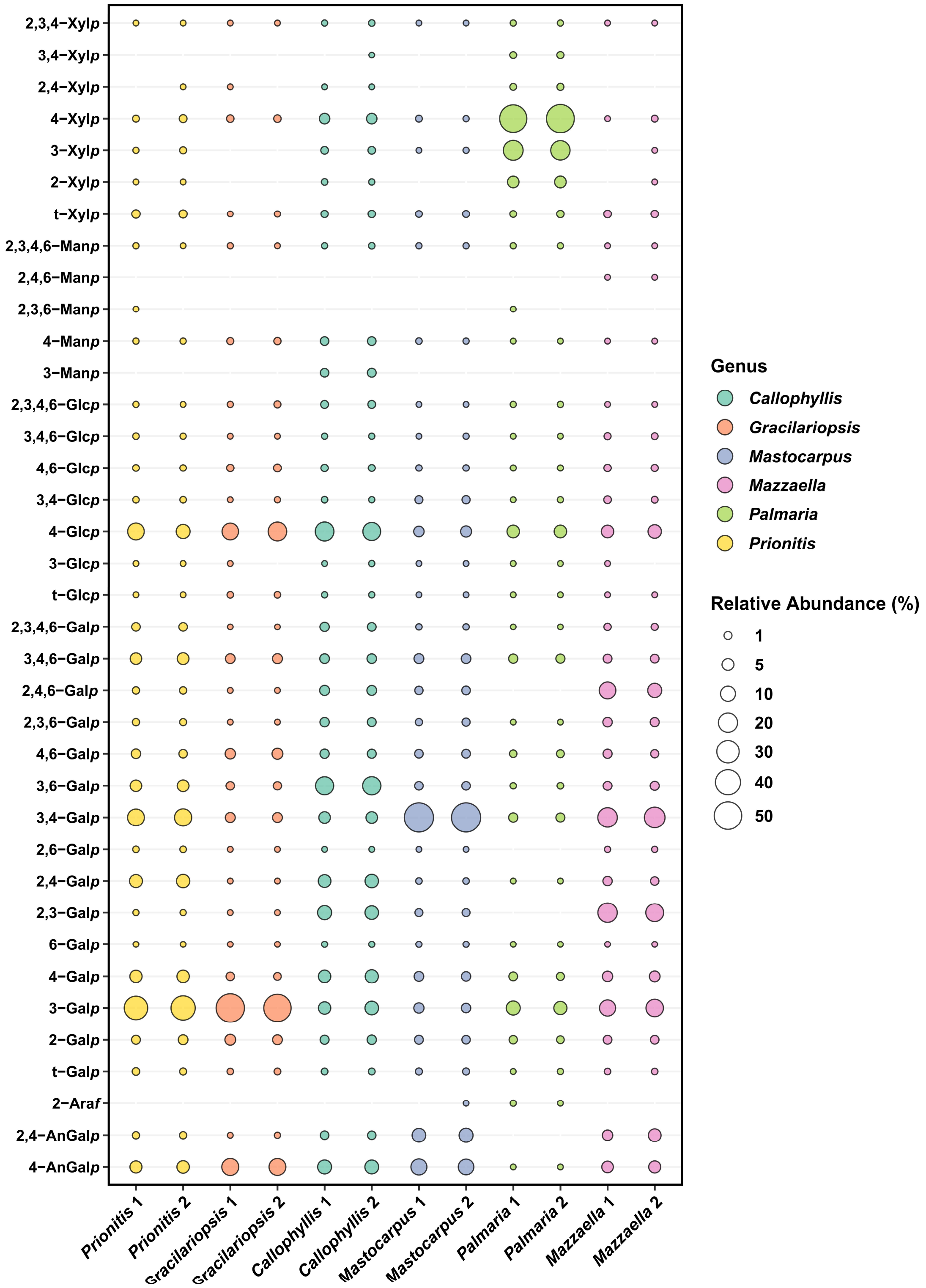

Figure S16. Bubble plot showing the relative linkage composition (Mol%) quantified by GC-MS from each of the two separate experiments conducted on unfractionated polysaccharides from six red seaweed species.

R Script code for bubble plot:

```
# Load required libraries
library(ggplot2)   # For creating data visualizations
library(reshape2)  # For data reshaping and transformation

# Insert the name of your CSV file here.
# Read data from the CSV file and prevent modification of column names
pc <- read.csv("File Name.csv", header = TRUE, check.names = FALSE)

# Melt the data to long format for ggplot2.
# Replace "Sample" and "Genus" in the entire script with the names of the first 2 columns of your CSV file.
# The 2nd column should be the treatment group, harvest year, disease state, etc.
pcm <- melt(pc, id = c("Sample", "Genus"))
pcm$Sample <- factor(pcm$Sample, levels = unique(pcm$Sample))

# Create the bubble plot
plot <- ggplot(pcm, aes(x = Sample, y = variable)) +
  geom_point(aes(size = value, fill = Genus), alpha = 0.75, shape = 21) +
  scale_size_continuous(limits = c(0.000001, 100), range = c(1, 10), breaks = c(1, 5, 10, 20, 30, 40, 50)) + #limits sets min and max values for your data set, range sets size of bubbles
  labs(x = "", y = "", size = "Relative Abundance (%)", fill = "Genus")  +
  theme(
    legend.key = element_blank(),
    axis.text.x = element_text(colour = "black", size = 10, face = "bold", angle = 45, vjust = 1, hjust = 1),  # Adjust angle, horizontal (hjust), and vertical alignment (vjust) of x-axis labels
    axis.text.y = element_text(colour = "black", face = "bold", size = 9),
    legend.text = element_text(size = 10, face = "bold", colour = "black"),
    legend.title = element_text(size = 11, face = "bold"),
    panel.background = element_blank(),
    panel.border = element_rect(colour = "black", fill = NA, size = 1.2),
    legend.position = "right",
    panel.grid.major.y = element_line(colour = "grey95")
  ) +
  guides(fill = guide_legend(override.aes = list(size = 4)))+ # Set size of upper legend labels
  scale_fill_brewer(palette = "Set2")  # Use the Set2 color palette for the bubbles

#Display the plot on RStudio
print(plot)

#Set output file name and save the bubble plot as a PDF or PNG image and change the size of the image
ggsave("File Name.pdf", plot = plot, width = 8, height = 11, units = "in", dpi = 1200)
```

R Script code for PCA score plot:

```
# Load required libraries
library(dplyr)  # For data manipulation
library(forcats)  # For further data manipulation
library(ggplot2)  # For plotting

# Insert the name of your file here
data <- read.csv("File Name.csv")

# Perform principal component analysis (PCA), scale=true will subtract mean and divide by SDev
pca <- prcomp(data[, -1], scale = TRUE)

# Create a data frame with PCA results
pca_data <- as.data.frame(pca$x[, 1:2])
pca_data$Label <- data[, 1]

# Display summary of PCA results
summary(pca)

biplot(pca ,scale=0)

# Extract PC scores
str(pca)
pca$x
data2 <- cbind(data, pca$x[, 1:2])
head(data2)

# Extract variance explained by each principal component
variance_explained <- round(pca$sdev^2 / sum(pca$sdev^2) * 100, 2)

# Format variance explained to show 1 significant figure
variance_explained <- sprintf("%.1f", variance_explained)

# Create PCA plot using ggplot2
pca_plot <- ggplot(pca_data, aes(x = PC1, y = PC2, color = Label, fill = Label)) +
  geom_point(size = 5, shape = 16) +
  stat_ellipse(alpha = 0.2) +  # Add ellipses
  labs(
    x = paste("PC1, (", variance_explained[1], "%)", sep = ""),   # X-axis label
    y = paste("PC2, (", variance_explained[2], "%)", sep = ""),   # Y-axis label
    color = expression("Genus"),  # Set the legend title for the color aesthetic
    fill = expression("Genus")    # Set the legend title for the fill aesthetic
  ) +
  guides(
    color = guide_legend(override.aes = list(shape = 16, size = 4)), #legend label shape and size
    fill = guide_legend(override.aes = list(shape = 16, size = 4))  #legend label shape and size
  ) +
  theme_minimal() +
  theme(
    legend.key.size = unit(0.7, "cm"),  # Adjust the size of legend keys
    legend.text = element_text(size = 12, face = "italic"),  # Adjust the size of legend text, and make it italic
    legend.title = element_text(size = 14),  # Adjust the size of legend title
    plot.background = element_rect(fill = "white"),  # Set plot background color
```

```
    plot.title = element_text(size = 16, hjust = 0.5),  # Adjust plot title size and center it
    axis.text = element_text(size = 12),  # Adjust the font size for axis tick labels
    axis.title = element_text(size = 14),  # Adjust the font size for axis labels
    panel.grid.major = element_line(color = "gray", size = 0.5),  # Add major gridlines
    panel.grid.minor = element_blank(),  # Remove minor gridlines
    panel.border = element_rect(color = "black", fill = NA, size = 1)  # Add black border
  ) +
  ggtitle("")  # Add a title to the plot

# Update the color palette from ColorBrewer
pca_plot <- pca_plot +
  scale_color_brewer(palette = "Set2")  # Change the color palette to any set in ColorBrewer

pca_plot <- pca_plot +
  scale_fill_brewer(palette = "Set2")  # Change the color palette for ellipses and legend

# Display the plot
print(pca_plot)

# Set output file name and save PCA plot as PDF or PNG image and change the size of the image
ggsave("File Name.png", plot = pca_plot, width = 10, height = 8, units = "in", dpi = 900)
```
